# SLIT3-mediated intratumoral crosstalk induces neuroblastoma differentiation via a spontaneous regression-like program

**DOI:** 10.1186/s12967-025-06621-0

**Published:** 2025-05-30

**Authors:** Meiling Liu, Dekang Lv, Wenjing Yan, Yi Wu, Shulan Wang, Luoxuan Wang, Jie Lei, Deshun Zeng, Zifeng Wang, Fang Liu, Bing Deng, Quentin Liu, Bin He, Min Yan

**Affiliations:** 1 State Key Laboratory of Oncology in South China, Guangdong Provincial Clinical Research Center for Cancer, Psychobehavioral Cancer Research Center, Sun Yat-Sen University Cancer Center, Guangzhou, 510060 People’s Republic of China; 2 Institute of Cancer Stem Cell, Dalian Medical University, 9 West Section, Lvshun South Road, Dalian, 116044 Liaoning China

**Keywords:** Neuroblastoma, Intra-tumor cell heterogeneity, SLIT3, SLIT-ROBO signaling, Differentiation induction therapy

## Abstract

**Background:**

Neuroblastoma, the most common pediatric extracranial solid tumor, has heterogeneous clinical outcomes ranging from malignant progression to spontaneous regression. With the highest frequency of the elusive spontaneous regression, low-risk INSS Stage 4S neuroblastoma represents an ideal model for mechanistic investigation. Spontaneous regression is often accompanied by tumor differentiation, but the mechanisms underlying this process remain largely unclear.

**Methods:**

Single-nucleus transcriptomics (snRNA-seq) data of neuroblastoma samples were obtained from the Synapse repository to investigate the composition of heterogeneous tumor cell clusters. The feature of the Stage 4S-specific tumor cell subpopulation was revealed through differential expression analysis, pathway enrichment analysis and pseudotime analysis, followed by clinical significance validation on public cohort datasets. The biological function of secreted SLIT3 was validated using multiple in vitro models, including recombinant protein treatment, conditioned medium treatment, and cell lines coculture, to confirm the intratumoral crosstalk effect. Orthotopic and subcutaneous xenograft models were established to verify SLIT3's in vivo function. Cellular bulk RNA-seq analysis was performed with or without SLIT3 recombinant protein treatment to discover the downstream pathways activated by SLIT3, followed by validation with specific pathway inhibitors.

**Results:**

Analysis of snRNA-seq revealed a distinct subpopulation of tumor cells within INSS Stage 4S neuroblastoma, characterized by a spontaneous regression-like program progressing toward differentiation. Activated SLIT-ROBO signaling was found in the Stage 4S-specific tumor cell subpopulation, which strongly correlated with favorable prognosis. Further investigation into the secreted ligands in SLIT-ROBO related pathways revealed that SLIT3 displayed the most potent enrichment in Stage 4S tumors and the strongest differentiation-inducing effect. In vitro experiments using recombinant SLIT3 protein, conditioned medium, and cell lines coculture consistently demonstrated the capacity of SLIT3 to induce neuroblastoma cell differentiation via intratumoral crosstalk, as evidenced by increased neurite outgrowth and elevated expression of neuronal differentiation markers. Both orthotopic xenograft and subcutaneous xenograft models demonstrated that SLIT3 expression suppressed tumor growth, leading to in vivo tumor differentiation. Mechanistically, PLCβ/PKC signaling mediates the SLIT3-induced neuroblastoma cell differentiation.

**Conclusions:**

Stage 4S-specific tumor cell subpopulation exhibits a spontaneous regression-like program, from which SLIT3 mediates intratumoral crosstalk and promotes neuroblastoma differentiation via PLCβ/PKC signaling. These findings provide new insights into the mechanism of spontaneous regression in neuroblastoma and offer novel therapeutic targets for differentiation-based treatment strategies.

**Supplementary Information:**

The online version contains supplementary material available at 10.1186/s12967-025-06621-0.

## Background

Neuroblastoma, originating from the neural crest lineage, accounts for 15% of all childhood cancer-related deaths [1, 2]. Neuroblastoma displays remarkably heterogeneous clinical manifestations, ranging from highly aggressive malignant growth to spontaneous regression. Based on the International Neuroblastoma Staging System (INSS) classification [3], low-risk Stage 4S tumors have a favorable prognosis referring to a 5-year overall survival (OS) reaching 90% and a 69% probability of spontaneous regression [4–6]. On the other hand, high-risk Stage 4 tumors have a 5-year OS less than 50%, with half cases developing primary treatment resistance or relapse [7, 8]. Mechanism investigation into spontaneous regression will provide new opportunities for high-risk neuroblastoma therapy. However, the underlying mechanism remains to be elucidated.

Spontaneous regression refers to the reduction in size or complete disappearance of a primary tumor or metastatic lesion, with minimal or no therapeutic intervention [9, 10]. This phenomenon has been reported in various types of tumors, but is most frequently observed in neuroblastomas [9, 11, 12]. Several possible mechanisms have been proposed to explain spontaneous regression, including tumor cell apoptosis and immune-mediated clearance [13]. Tumor cell apoptosis can be induced by neurotrophins deprivation, lack of telomere maintenance mechanisms, and HOXC9 expression [13–15]. Tumor-targeted T cells and antineuronal antibodies were found in certain patients, indicating potential immune clearance in neuroblastoma; nevertheless, more evidence is needed to support these findings [16, 17]. On the other hand, spontaneous regression is frequently associated with tumor differentiation. Mature ganglion cells emerge within the low-risk tumor over time, and the tumor may finally differentiate into a benign ganglioneuroma [9, 18]. These observations suggest the presence of unique intrinsic regulatory program, driving tumor differentiation and subsequent regression in neuroblastoma. Unraveling the intratumoral heterogeneity is crucial for elaborating the intrinsic regulatory program within neuroblastoma. Heterogeneity in tumor cells contributes to diverse tumor behaviors, including therapeutic resistance driven by drug-resistant cells and tumor recurrence mediated by tumor stem cells [19–21]. Extensive heterogeneous clinical outcomes indicate noteworthy intratumoral heterogeneity in neuroblastoma [22, 23]. Whether a particular tumor cell population is involved in driving benign tumor behaviors, such as spontaneous regression, remains an open question.

SLIT3 is a large extracellular matrix-secreted glycoprotein belonging to the SLIT family and serving as a major ligand of the SLIT-ROBO signaling pathway [24]. The SLIT-ROBO signaling pathway typically participates in axon guidance during nervous system development, and has also been reported to regulate neuron differentiation [25–27]. More recently, its tumor-suppressive properties, including inhibition of tumor cell proliferation and migration, have been reported in breast cancer and lung cancer [28–30]. Notably, SLIT2 acts as a migration inhibitor in neuroblastoma, whose promoter is found to be extensively methylated [31, 32]. These findings suggest a potential role of SLIT-ROBO signaling pathway acting as a promising target for neuroblastoma therapy. Due to the absence of autocatalytic or enzymatic activity, activated ROBO receptors rely on scaffolding molecules and downstream signaling, such as Abelson tyrosine kinase (Abl), Slit-Robo-GTPase activating proteins (srGAPs), or Wnt signaling, to exert their regulatory functions [24, 29]. However, the roles and downstream mechanisms of SLIT3 and SLIT-ROBO signaling in the context of neuroblastoma remain largely unexplored.

In this study, by employing an available neuroblastoma single-nucleus transcriptome data [33], we identified an intrinsic spontaneous regression-like program within a distinct tumor cell subpopulation from Stage 4S tumors. This subpopulation of tumor cells is characterized by high levels of differentiation, and activated SLIT-ROBO signaling pathways that are associated with favorable prognosis. Further experimental investigation demonstrated that SLIT3 conveys intratumoral crosstalk leading to neuroblastoma differentiation in vitro and in vivo. Mechanistically, PLCβ/PKC signaling mediates SLIT3-induced neuroblastoma cell differentiation. Taken together, our results uncover a new mechanism underlying neuroblastoma spontaneous regression, and provide novel targets for differentiation-inducing therapy in high-risk neuroblastoma.

## Methods

### Cell culture

The neuroblastoma cell lines SK-N-SH, SK-N-BE(2) and Kelly used in this study were obtained from ATCC (https://www.atcc.org/). Cells were maintained in RPMI 1640 (Gibco) supplemented with 10% (v/v) fetal bovine serum (FBS) (HyClone). The human embryonic kidney cell line 293T was maintained in DMEM (Gibco) supplemented with 10% (v/v) FBS. All cell lines were maintained at 37 °C incubator containing 5% CO_2_. Cells were passaged at 80–90% confluence, and culture medium was changed every 3 days. All cell lines were regularly tested for mycoplasma contamination.

### Vector construction

Expression vectors for NCAM1, NRCAM, VSTM2L, SLIT2, SLIT3, NECTIN1, ALCAM, NRP1, and NRP2 were constructed using pcDNA6/myc-His backbone. Coding sequences were obtained from commercial plasmids or synthesized from cell line mRNA using One-Step gDNA Removal and cDNA Synthesis SuperMix (TransGen). The sequences were inserted into the backbone by homologous recombination using One Step Cloning Kit (Vazyme). pLenti6-3Flag (Ctrl-3F) and pLenti6-SLIT3-3Flag (SLIT3-3F) vectors were constructed from pLenti6/V5-Dest backbone by replacing the V5 tag with a 3×Flag tag. Doxycycline-inducible vectors pLVX-TRE-3Flag (TRE-3F) and pLVX-TRE-SLIT3-3Flag (TRE-SLIT3-3F) were constructed from pLVX-TRE3G-IRES backbone.

### Transfection and lentiviral transduction

For transient transfection, polyethylenimine (PEI) was used as the transfection reagent at a DNA:PEI ratio of 1:3 (w/w). Target cells were transfected at a confluence of 30–50%. The culture medium of transfected cells was replaced 12 h post-transfection. For stable cell line establishment, lentiviral particles were produced in 293T cells. Briefly, vectors were co-transfected with packaging plasmids at a ratio of vector:pMD2.G:psPAX2 = 1:3:4 using PEI. The culture medium was replaced 12 h post-transfection, and virus-containing supernatants were collected at 48 h and 72 h, filtered through a 0.45-μm filter, and stored at −80 °C. For lentiviral transduction, viral supernatants were thawed and added to target cells supplemented with 8 μg/mL polybrene. Culture medium was changed 24 h post-transduction, and stable cells were selected with appropriate antibiotics subsequently.

### Morphological determination of cell differentiation

For differentiation induction, cells were seeded at a density of 10^5^ cells/mL in 6-well plates 24 h prior to treatment with recombinant SLIT3 protein. After 72 h treatment with or without 200 ng/mL recombinant SLIT3 protein, cell morphology was documented by capturing bright-field images from three independent experiments using a Nikon ECLIPSE Ti2 inverted fluorescence microscope. The longest axis of the observed cell body was measured from the morphology images using ImageJ software. For statistical analysis, more than 20 cells were evaluated for each group from five random fields of view.

### Conditioned medium

293T cells stably expressing SLIT3-3Flag (pLenti6-SLIT3-3Flag) or Ctrl vector (pLenti6-3Flag) were used to generate conditioned medium. When cells reached approximately 80% confluence in 10-cm culture dishes, the complete medium was replaced with serum-free medium. After 24 h, the cell culture supernatant was collected, filtered through a 0.45-μm filter, aliquoted, and stored at −80 °C. For experiments, conditioned medium was thawed and diluted 1:2 with fresh culture medium, supplemented with FBS to a final concentration of 10% (v/v). Target cells were then cultured in this conditioned medium mixture.

### Cell lines coculture

Stable doxycycline-inducible donor cell lines expressing pLVX-TRE-3Flag (TRE-3F) or pLVX-TRE-SLIT3-3Flag (TRE-SLIT3-3F) were established in SK-N-BE(2) and SK-N-SH cells stably containing pLVX-Tet3G vector. Wild-type recipient cells were labeled with mNeonGreen (mNG) fluorescent protein using pLenti6-mNeonGreen vector. For coculture experiments, mNG-positive recipient cells were mixed with TRE-3F (Ctrl group) or TRE-SLIT3-3F (SLIT3 group) donor cells at a ratio of 7:3. The cell mixture was seeded into 10-cm culture dishes at a density of 1 × 10^6^ cells per dish. After 5 days of coculture in the presence of 1 μg/mL doxycycline, cells were harvested and sorted by fluorescence-activated cell sorting (FACS). Top 30% mNG-positive recipient cells were collected for subsequent assays.

### Western blot analysis

Total proteins were extracted using RIPA lysis buffer (GBCBIO) supplemented with protease and phosphatase inhibitors (TargetMol). Protein concentrations were determined by Bradford assay. Equal amounts of protein (20 μg per lane) were separated by SDS-PAGE and transferred onto 0.45-μm PVDF membranes. The membranes were blocked with 5% BSA in TBST for 1 h at room temperature, followed by overnight incubation with primary antibodies at 4 °C. The following primary antibodies were used: rabbit anti-pTrkA (PTMBio), rabbit anti-MAP2 (Proteintech), mouse anti-SNAP25 (PTMBio), rabbit anti-SLIT3 (Affinity), mouse anti-FLAG (Sigma-Aldrich) and mouse anti-GAPDH (Proteintech). After washing with TBST, membranes were incubated with HRP-conjugated secondary antibodies: goat anti-mouse IgG (Invitrogen) or goat anti-rabbit IgG (Invitrogen). Protein bands were visualized using a chemiluminescence detection kit (EpiZyme). Each presented experiment was performed three times independently. Densitometry measurements of the protein bands were carried out using ImageJ according to the prior double normalization protocol [34, 35]. In brief, protein expression levels were first normalized to the internal reference protein (e.g., GAPDH), and then presented as fold changes relative to the control group. For proteins that showed no detectable expression in the control group, normalization was performed against the internal reference protein.

### Quantitative reverse transcription polymerase chain reaction (RT-qPCR)

Total RNA was extracted from cells using EZ-press RNA Purification Kit (EZBioscience) according to the manufacturer's instructions. First-strand cDNA was synthesized using TransScript All-in-One First-Strand cDNA Synthesis SuperMix (TransGen). RT-qPCR was performed using SYBR qPCR Master Mix (Vazyme). Each presented experiment was performed three times independently. Primer sequences were obtained from PrimerBank (https://pga.mgh.harvard.edu/primerbank/) and listed in Supplementary Table S1. The relative gene expression was calculated using the 2^-ΔΔCt^ method with *ACTB* as the internal control [36].

### Xenograft models

For orthotopic adrenal xenografts, 12 male BALB/c nude mice (4–8 weeks old) were obtained from GemPharmatech Biotechnology Co., Ltd. (Jiangsu, China) and divided into two groups randomly. SK-N-SH cell lines for xenograft model were stably labelled with pLenti6-luciferase. Donor cells containing pLVX-TRE-3Flag (TRE-3F) or pLVX-TRE-SLIT3-3Flag (TRE-SLIT3-3F) were mixed with wild-type recipient cells at a ratio of 3:7. The cell mixture was diluted 1:1 with Matrigel to a final concentration of 8 × 10^7^ cells/mL. Under surgical procedure with isoflurane anesthesia, 25 μL of cell suspension was injected into the site of adrenal gland. The surgical incision was closed with absorbable sutures subsequently.

For subcutaneous xenografts, 10 male NOD-SCID mice (4–8 weeks old) were obtained from GemPharmatech Biotechnology Co., Ltd. (Jiangsu, China). SK-N-SH cell mixtures were prepared as described above, but diluted to 2 × 10^7^ cells/mL. Ctrl group and SLIT3 group cells (100 μL each) were injected subcutaneously into the left and right flanks of NOD-SCID mice, respectively.

Mice with xenograft were administered doxycycline (2 mg/mL) in drinking water immediately after implantation to induce SLIT3 expression. Regular body weight measurements were conducted to monitor general health status. Bioluminescence signaling was detected by intraperitoneal injection of D-luciferin (YEASEN) in a dosage of 1.5 mg per 20 g body weight. Tumor growth was monitored by bioluminescence imaging using an IVIS imaging system. All animal procedures were approved by the Institutional Animal Care and Use Committee of Sun Yat-sen University Cancer Center and conducted in accordance with the 3Rs principles.

### Histological analysis

Tumor samples from subcutaneous xenografts were collected and fixed in 4% paraformaldehyde prior to embedding in paraffin. Embedded samples were cut at 4 μm thickness and stored at –80 °C. For hematoxylin and eosin (H&E) staining, sections were deparaffinized, rehydrated through graded alcohols, and stained with hematoxylin (3 min) and eosin (1 min) using an H&E staining kit (Biosharp) according to the manufacturer's instructions. The stained sections were dehydrated through graded alcohols, cleared in xylene, and mounted with coverslips. Images were captured using a NIKON ECLIPSE Ni microscope.

For immunohistochemical analysis, tumor sections were deparaffinized and rehydrated following the same protocol as H&E staining. Antigen retrieval was performed under high pressure using 0.01 M sodium citrate buffer (pH 6.0). The sections were then permeabilized with 0.5% Triton X-100 and treated with 3% hydrogen peroxide to quench endogenous peroxidase activity. After blocking with goat serum, sections were incubated with primary antibodies overnight at 4 °C. The following primary antibodies were used: rabbit anti-SLIT3 (Affinity), rabbit anti-CHGA (Proteintech), and mouse anti-SNAP25 (PTMBio). The next day, after washing with PBS, sections were processed with a mouse/rabbit polymer detection system (ZSbio) for visualization of DAB. The stained sections were then dehydrated, cleared, mounted, and imaged following the same protocol as H&E staining. Images were captured using a NIKON ECLIPSE Ni microscope. Quantification of DAB signal intensity was performed using IHC-profiler plugin in ImageJ software. Statistical analyses were performed using data collected from 5 random fields of view for each protein per group.

### snRNA-seq data analysis

Available single-nucleus RNA sequencing data generated using Smart-seq2 protocol were obtained from the Synapse repository (Project ID: syn22302605, https://www.synapse.org) upon reasonable request. Raw sequencing data were quality-controlled using FastQC, followed by adapter trimming using Trimmomatic. The cleaned reads were aligned to the human reference genome (GRCh37) using STAR, and gene expression was quantified using featureCounts to generate the gene expression matrix.

Subsequent analyses were performed with Seurat (v4.0.4) software. All data processing steps were performed using functions with default parameters implemented in Seurat (NormalizeData, FindVariableFeatures, ScaleData, RunPCA, FindNeighbours, FindClusters and RunTSNE). Cell type annotation was performed using the SingleR (v2.8.0) R package with HumanPrimaryCellAtlasData as reference dataset [37]. Chromosomal copy number variation (CNV) scores for each cell cluster were estimated using the inferCNV (v1.22.0) pipeline with object generated from the snRNA Seurat expression matrix by the CreateInfercnvObject function, and then were analyzed using inferCNV::run with default parameters [38]. Gene Set Variation Analysis (GSVA) was employed to assign gene set enrichment scores for cell clusters using the GSVA (v2.1.4) R package, with cluster expression matrix generated from the snRNA Seurat object and gene sets from the GO: Biological Process category of the MSigDB database.

The ordering genes used for pseudotime analysis were generated from the differentialGeneTest function and Branched Expression Analysis Modeling (BEAM) function from the monocle (v2.34.0) R package [39]. Cells were ordered according to the ordering genes using the orderCells function. Pseudotime trajectory was generated using the plot_cell_trajectory function and the plot_genes_branched_pseudotime function. Differential gene expression analysis was conducted between tumor cell clusters cIVS and cIV using the FindMarkers function in Seurat. Gene Set Enrichment Analysis (GSEA) was performed on the differentially expressed genes using the gsePathway function from the clusterProfiler (v4.14.6) R package and gene sets from the Reactome database. Gene Ontology (GO) enrichment analysis for overlapping secretome genes was performed on The Gene Ontology Resource (https://geneontology.org/). Intercellular communication between the cell clusters were identified by CellPhoneDB (v5.0.1) supplemented with additional curated ligand-receptor pairs [40].

### Cellular bulk RNA-seq data analysis

Total RNA for bulk RNA-seq was extracted from SK-N-BE(2) cells with or without SLIT3 treatment using EZ-press RNA Purification Kit (EZBioscience). Library construction and RNA sequencing were performed by Novogene Co., Ltd. (Beijing, China) on an Illumina HiSeq2000 platform with 150 bp paired-end reads. The raw sequencing data were trimmed and quality-controlled using FastQC, followed by alignment and differential gene expression analysis using the RNA Cocktail framework [41]. GSEA was performed on the differentially expressed genes using the clusterProfiler R package. The gsePathway function was applied with gene sets from the Reactome database, while the gseGO function was applied with gene sets from the Gene Ontology Biological Process database.

### Public database analysis

Survival analysis and gene set scoring in multiple neuroblastoma cohorts were performed at the R2: Genomics Analysis and Visualization Platform (http://r2.amc.nl). Gene set scores were calculated as z-scores of gene expression within each gene set. Sample staging information for each cohort was obtained from the R2 platform. Correlation analysis of gene set scores was performed using built-in functions in R. Receiver Operating Characteristic (ROC) analysis was conducted using the multipleROC R package.

### Statistical analysis

Statistical analysis was performed using R software (https://www.r-project.org/). Comparison between two groups was carried out using Student’s t-test. Comparison among multiple groups was carried out using one-way ANOVA analysis. Quantitative results were presented as the mean ± SEM. *p* < 0.05 was regarded as statistically significant.

## Results

### A distinct tumor cell subpopulation exists in Stage 4S neuroblastoma

**Fig. 1:**
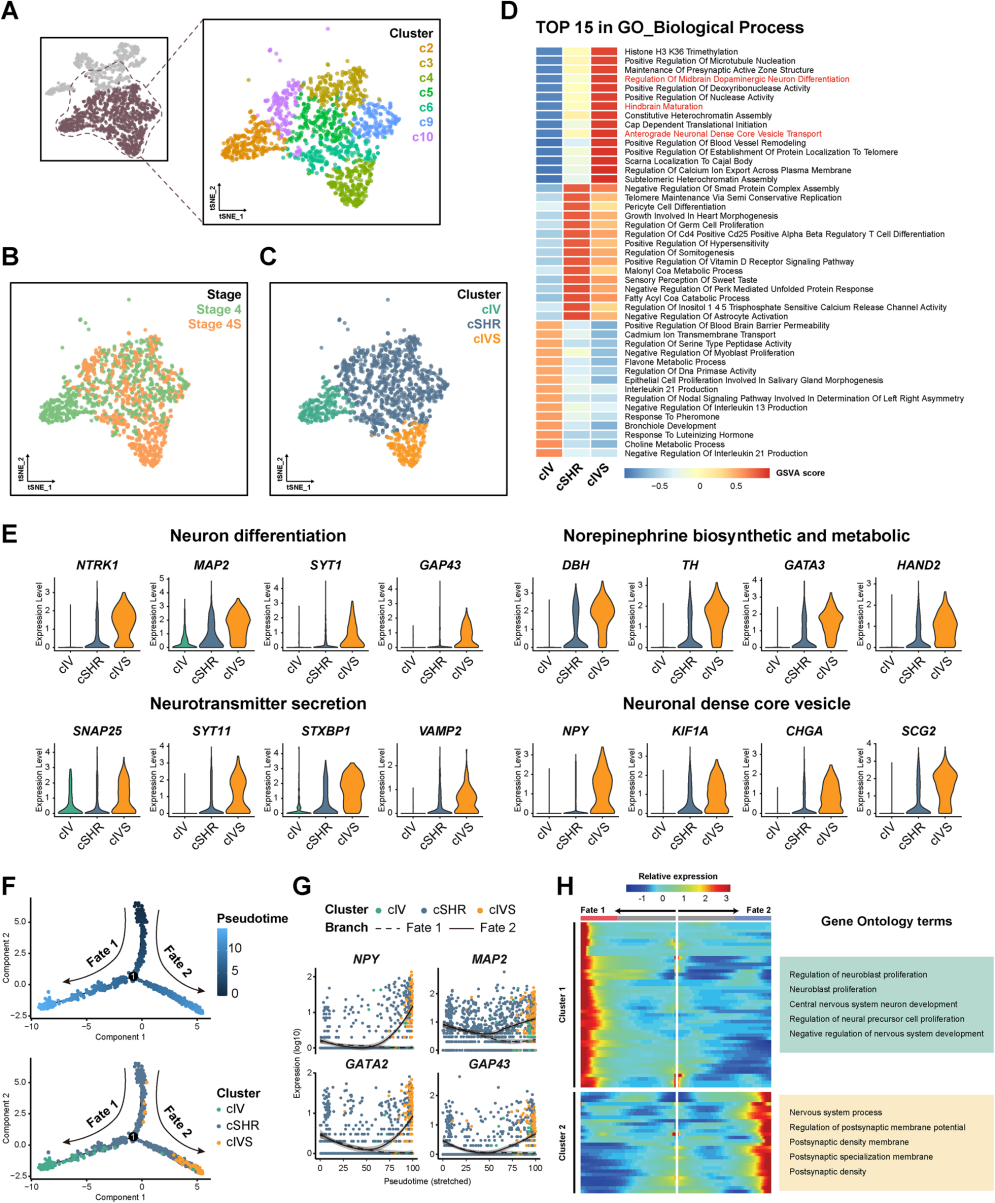
A distinct tumor cell subpopulation exists in Stage 4S neuroblastoma. **A** tSNE visualization displaying identified tumor cell clusters selected for subsequent analyses. **B** tSNE visualization of identified tumor cells colored by disease stage. **C** tSNE visualization of identified tumor cells colored by stage-specific clusters. **D** Heatmap showing GSVA gene sets enrichment scores of the top 15 pathways from GO biological process database in clusters cIV, cSHR and cIVS. **E** Violin plots showing neuronal function markers expression across clusters cIV, cSHR and cIVS. **F** Pseudotime analysis of the identified tumor cells reveals two distinct cell fate trajectories (upper panel). Distribution of cells from clusters cIV, cSHR, and cIVS along the pseudotime trajectory (lower panel). **G** Pseudotemporal expression progressions of neuronal function markers associated with differentiating neuroblastoma along the two cell fate trajectories. **H** Left panel: Heat map showing the expression of branch-dependent genes identified by BEAM. The middle of the heat map shows pre-branch cells (gray), with the left-side representing cells from Fate 1 (red), and the right-side representing cells from Fate 2 (blue). Color intensity indicates relative gene expression levels. Right panel: GO terms enriched from BEAM-identified genes from cluster 1 (corresponding to Fate 1) and cluster 2 (corresponding to Fate 2), respectively

To investigate cellular heterogeneity in neuroblastoma, we requested the available snRNA-seq data from the Synapse repository [33]. Smart-seq2 snRNA-seq data comprising INSS Stage 4 and Stage 4S tumor samples were processed and filtered based on quality metrics, yielding 4,195 high-quality nuclei. Unsupervised clustering analysis followed by tSNE dimensionality reduction identified 12 clusters from the filtered nuclei (Figure S1 A-C). Stage 4S cells constituted a large proportion of clusters c4 (97.4%), while Stage 4 cells constituted the major proportion of multiple clusters including c1 (89.6%), c2 (96.8%), c7 (100%), c8 (100%), c11 (97.4%) and c12 (100%) (Figure S1 D). Cell type annotation using SingleR identified clusters c7 and c12 as endothelial cells and c11 as monocytes/macrophages, with elevated expression of representative cell type markers (Figure S1 E, F). Clusters c1, and c8 exhibit multiple mesenchymal cell identities and absent neuronal signatures, implicating their somatic cell identity. Clusters c2, c4, c5, c6, c9, and c10 showed strong neuronal signatures and enhanced neuronal markers expression, suggesting their tumor cell identity (Figure S1 E, F). To further distinguish tumor cells from diploid somatic cells, we performed inferCNV analysis to calculate cluster chromosome copy number variation (CNV) among clusters [38]. Using non-malignant clusters (c1, c7, c8, c11, and c12) as reference populations, the remaining clusters exhibited varying degrees of copy number alterations (Figure S1 G, H). The clusters with high CNV score and obvious neuronal features were selected as tumor cell clusters for subsequent analyses (Fig. 1A).

Based on the original sample stage, Stage 4 and Stage 4S cells showed distinct distribution patterns across clusters (Fig. 1B). We annotated the tumor cell clusters according to the stage proportion into three major cluster types: Stage 4S-enriched cluster (cIVS) from cluster c4, Stage 4-enriched cluster (cIV) from cluster c2, and shared clusters (cSHR) from remaining clusters containing cells from both stages (Fig. 1C). GSVA analysis revealed the enrichment of neural function-related pathways in cluster cIVS, indicating a more differentiated status of cIVS (Fig. 1D). Consistently, cluster cIVS exhibited higher expression of neuronal function markers compared to cluster cIV and cSHR (Fig. 1E). These results revealed a distinct tumor cell subpopulation within Stage 4S tumors, which exhibited higher differentiation levels. To further investigate the dynamic differences in cell differentiation states between clusters cIVS and cIV, pseudotime analysis was performed to reconstruct the developmental trajectory [39]. Analysis results revealed a branch point segregating two distinct fate endpoints of tumor cells, represented by cIV and cIVS (Fig. 1F). Fate 2, corresponding to cluster cIVS, exhibited increasing expression of neuronal function markers *NPY*, *MAP2*, *GAP43*, and *GATA2*, which are associated with differentiating neuroblastomas [42–45] (Fig. 1G). Gene Ontology (GO) analysis of branch-specific genes identified by BEAM analysis revealed enrichment of neuronal function terms and postsynaptic structure terms in Fate 2, while proliferation-related terms were enriched in Fate 1 (Fig. 1H). These results indicated that an intrinsic spontaneous regression-like program progressing toward differentiation exists within Stage 4S neuroblastoma.

### SLIT-ROBO signaling is activated in Stage 4S-specific tumor cell subpopulation

**Fig. 2:**
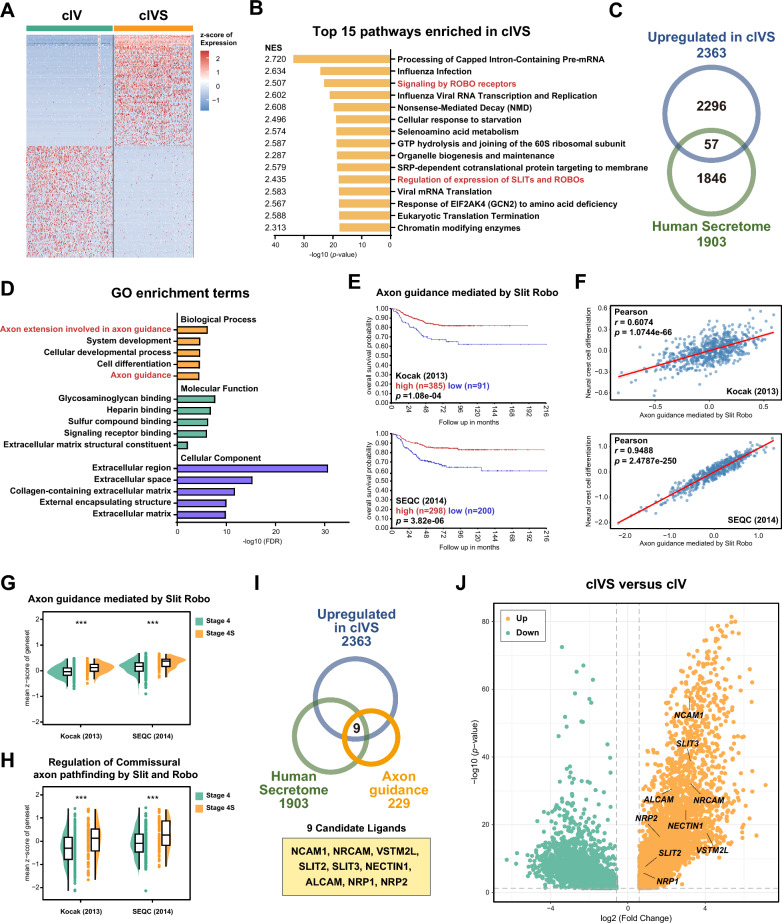
SLIT-ROBO signaling is activated in Stage 4S-specific tumor cell subpopulation. **A** Heatmap of DEGs between cIVS and cIV clusters. Analysis identified 4,903 DEGs (|FC|> 1.5, *p* < 0.05), including 2,363 upregulated and 2,540 downregulated genes. Top 200 upregulated and downregulated genes were shown. **B** Top 15 enriched pathways from Reactome database in cluster cIVS relative to cluster cIV. SLIT-ROBO signaling related pathways were highlighted in red. **C** Venn diagram depicting the intersection between upregulated genes in cIVS and The Human Secretome database, identifying 57 overlapping genes. **D** GO terms enrichment analysis of the 57 overlapping genes. The top 5 enriched terms in biological process, molecular function, and cellular component categories were shown. SLIT-ROBO signaling function-related axon guidance terms were highlighted in red. **E** Kaplan–Meier survival analysis based on the gene set score of"Axon guidance mediated by Slit Robo"in neuroblastoma cohorts Kocak (upper panel) and SEQC (lower panel). Patients were stratified into high (red) and low (blue) groups according to the gene set score. Statistical significance was determined by log-rank test. **F** Pearson correlation analysis among gene set scores in neuroblastoma cohorts Kocak (upper panel) and SEQC (lower panel). **G**, **H** Comparison of gene set scores in Stage 4 and Stage 4S samples from Kocak and SEQC cohorts. ***p < 0.001, Student’s t-test. **I** Venn diagram showing the identification of 9 candidate genes present in the gene set of Axon guidance from GO biological process database out of the 57 overlapping genes. **J** Volcano plot highlighting the 9 candidate genes upregulated in cluster cIVS relative to cluster cIV. Upregulated genes are colored in yellow and downregulated genes are colored in green

To identify the key factors contributing to the differentiated status of cluster cIVS, differential expression analysis was performed between cluster cIVS and cIV. 4,903 differentially expressed genes (DEGs) were identified (|FC|> 1.5, *p* < 0.05), among which 2,363 were upregulated and 2,540 were downregulated in cIVS (Fig. 2A). To identify the representative signaling pathways in the differentially expressed genes between clusters cIVS and cIV, Gene Set Enrichment Analysis (GSEA) was performed with gene sets from the Reactome database. SLIT-ROBO signaling related pathways were found among the top enriched pathways, including Signaling by ROBO receptors and Regulation of expression of SLITs and ROBOs (Fig. 2B). The SLIT-ROBO signaling has been well-established as a crucial pathway functioning in axon guidance during central nervous system development, and has been implicated in neural stem cell differentiation [46–49], while its functional role in neuroblastoma remains poorly characterized.

To further investigate the mechanism by which cluster cIVS affects the entire Stage 4S tumor, we examined the secretome profile associated with upregulated genes in this cluster. Among the 2,363 upregulated genes in cluster cIVS, we identified 57 genes encoding secreted proteins based on the Human Secretome Database [50] (Fig. 2C). GO enrichment analysis revealed axon guidance-related terms as the predominant biological process, including Axon Extension Involved in Axon Guidance and Axon Guidance (Fig. 2D). These findings aligned with the observed activation of SLIT-ROBO signaling in cluster cIVS and suggested intratumoral crosstalk launched by this cluster.

In both the Kocak cohort (n = 476) and SEQC cohort (n = 498) from R2 platform, patients with higher scores of SLIT-ROBO signaling-related gene sets exhibited significantly improved overall survival (Fig. 2E, Figure S2 A-C). Pearson correlation analysis revealed that the gene set score of SLIT-ROBO signaling was positively correlated with Neural crest cell differentiation, implying the intimate relationship between SLIT-ROBO signaling and differentiation in neuroblastoma, which originates from neural crest-derived precursor cells (Fig. 2F). Notably, from the bulk transcriptome data of neuroblastoma cohorts, low-risk Stage 4S tumors displayed significantly higher scores of SLIT-ROBO signaling-related gene sets compared with Stage 4 tumors (Fig. 2G, H). These results suggested potential clinical implications of SLIT-ROBO signaling in neuroblastoma.

**Fig. 3:**
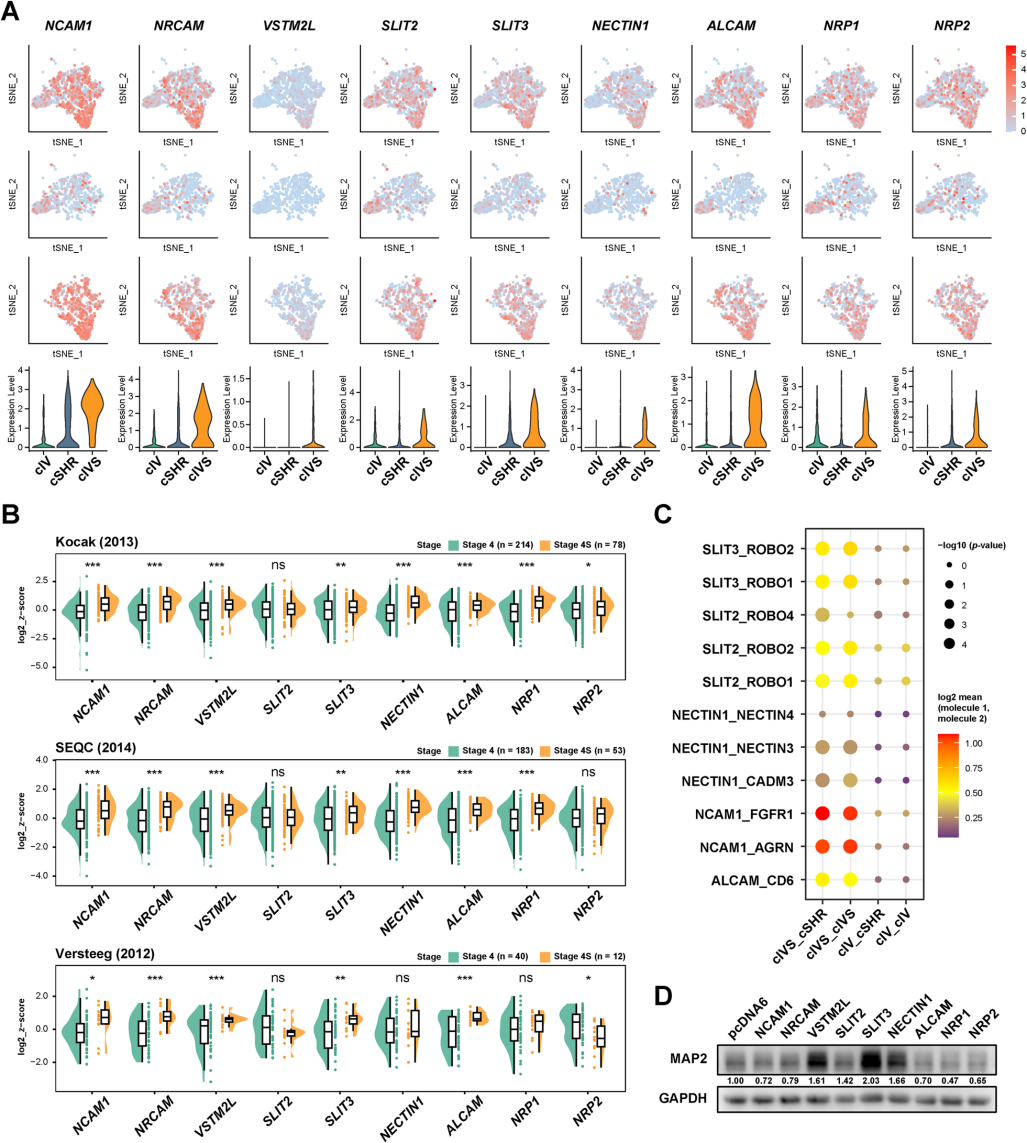
SLIT3 promotes neuroblastoma cell differentiation through intratumoral crosstalk. **A** tSNE visualization of the expression distribution for the 9 candidate genes across the stage-specific clusters (upper panel), with violin plots showing expression levels in each cluster (lower panel). **B** Half violin plots displaying expression of the 9 candidate genes in Stage 4S versus Stage 4 tumors from neuroblastoma cohorts including Kocak (upper panel), SEQC (middle panel), and Versteeg (lower panel). Gene expressions were presented as log2 of z-score. ns, not significant; *p < 0.05, **p < 0.01, ***p < 0.001, Student’s t-test. **C** CellPhoneDB analysis revealing the intratumoral crosstalk within clusters cIVS and cIV, as well as their respective interactions with the cluster cSHR. **D** Western blot analysis of the neuronal differentiation marker MAP2 in SK-N-SH cells following overexpression of the candidate genes

To identify the key driver of neuroblastoma differentiation within SLIT-ROBO signaling, 9 candidate genes (*NCAM1, NRCAM, VSTM2L, SLIT2, SLIT3, NECTIN1, ALCAM, NRP1,* and *NRP2*) were selected from the 57 upregulated secretome genes associated with axon guidance term (Fig. 2I, J). These candidates showed robust enrichment in cluster cIVS compared with cluster cIV (Fig. 3A). Nevertheless, *NCAM1*, *NRCAM*, *SLIT2*, *ALCAM*, *NRP1*, and *NRP2* also showed mild expression in Stage 4 tumor cells (primarily in cluster cIV). Meanwhile, three independent neuroblastoma cohorts (Kocak, SEQC, TARGET) consistently revealed significantly elevated expression of *NRCAM*, *VSTM2L*, *SLIT3*, and *ALCAM* in Stage 4S tumors (Fig. 3B). To further investigate the intratumoral crosstalk mediated by the candidates, CellPhoneDB was employed to systematically evaluate potential ligand-receptor interactions among the clusters [51]. Analysis results revealed that the interaction strength of SLIT-ROBO ligand-receptor pairs was significantly higher within Stage 4S tumors compared to Stage 4 tumors, from which we found considerable interaction between SLIT2/SLIT3 ligands and their cognate receptors ROBO1/ROBO2 (Fig. 3C). Nevertheless, the complete intercellular communication landscape was limited by the current database coverage of known interaction pairs. To validate the function of these candidate genes in neuroblastoma differentiation, the nine identified genes were overexpressed in the SK-N-SH neuroblastoma cell line. Among these candidates, SLIT3 led to the most significant upregulation of the neuronal differentiation marker MAP2, followed by moderate effects observed with VSTM2L and NECTIN1 (Fig. 3D). Given the robust differentiation-inducing capacity and consistent prominence in Stage 4S tumor and cluster cIVS, SLIT3 was selected as the primary candidate for subsequent functional validation.

### SLIT3 promotes neuroblastoma cell differentiation through intratumoral crosstalk

**Fig. 4:**
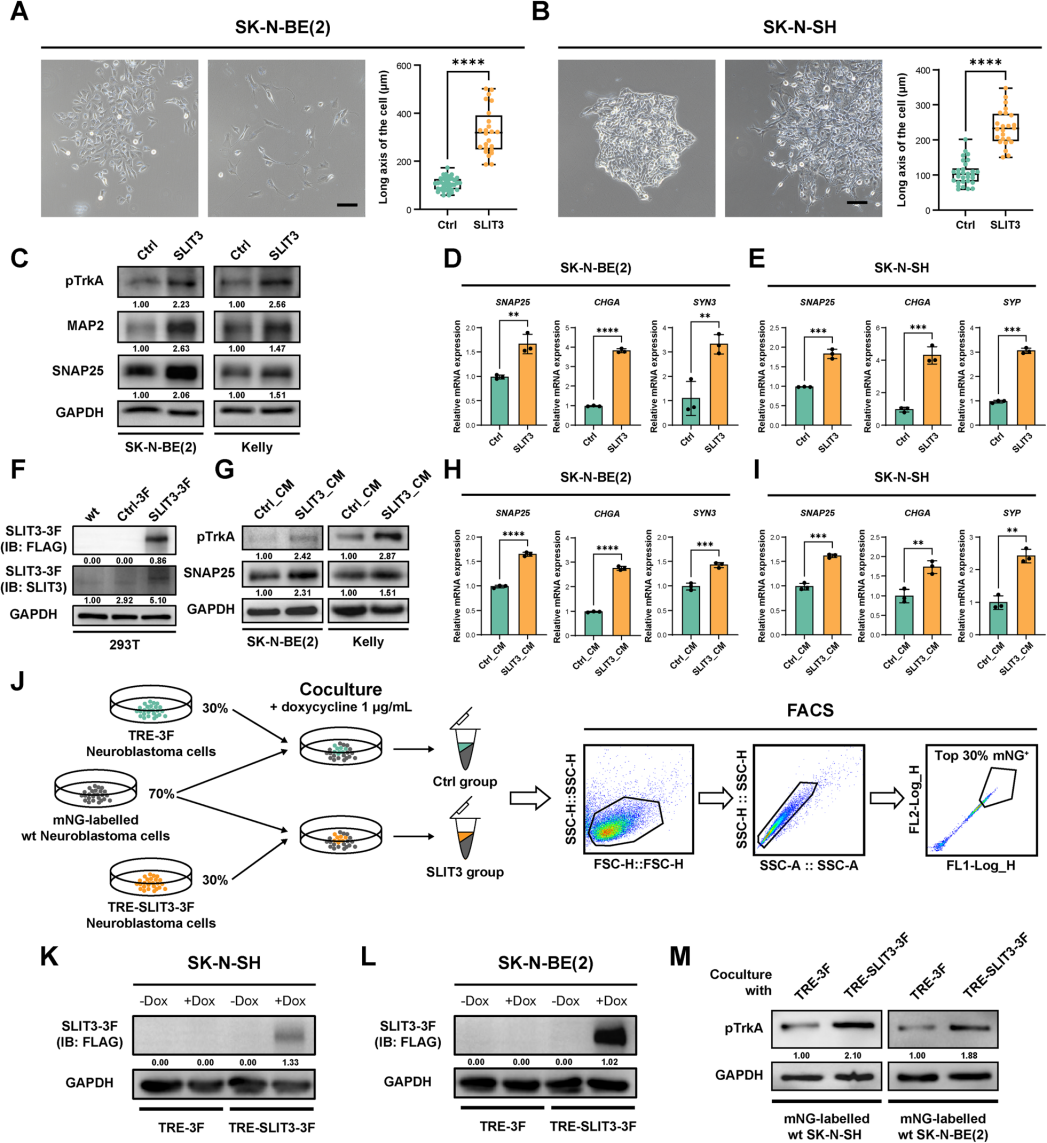
SLIT3 promotes neuroblastoma cell differentiation. **A** Representative images of SK-N-BE(2) cells treated with or without 200 ng/mL SLIT3 recombinant protein for 72 h, and statistical analysis of cell long axis length (n = 36 for Ctrl group and n = 24 for SLIT3 group, Student's t-test, ****p < 0.0001). Scale bars: 200 μm. **B** Representative images of SK-N-SH cells treated with or without 200 ng/mL SLIT3 recombinant protein for 72 h, and statistical analysis of cell long axis length (n = 30 for Ctrl and n = 24 for SLIT3, Student's t-test, ****p < 0.0001). Scale bars: 200 μm. **C** Western blot analysis of neuronal differentiation markers pTrkA, MAP2 and SNAP25 in SK-N-BE(2) cells and Kelly cells treated with or without 200 ng/mL SLIT3 recombinant protein for 72 h. **D** RT-qPCR analysis of neuronal differentiation marker *SNAP25*, *CHGA*, *SYN3* mRNA expression in SK-N-BE(2) cells treated with or without 200 ng/mL SLIT3 recombinant protein for 72 h. Data were presented as mean ± SD (n = 3, Student's t-test, **p < 0.01, ****p < 0.0001). **E** RT-qPCR analysis of neuronal differentiation marker *SNAP25*, *CHGA*, *SYP* mRNA expression in SK-N-SH cells treated with or without 200 ng/mL SLIT3 recombinant protein for 72 h. Data were presented as mean ± SD (n = 3, Student's t-test, ***p < 0.001). **F** Western blot analysis of SLIT3-3F expression in wild type (wt) and stable 293T cell lines (Ctrl-3F, pLenti6-3Flag; SLIT3-3F, pLenti6-SLIT3-3Flag) for conditioned medium production. **G** Western blot analysis of neuronal differentiation markers pTrkA and SNAP25 in SK-N-BE(2) cells and Kelly cells treated with control (Ctrl_CM) or SLIT3-containing (SLIT3_CM) conditioned medium. **H** RT-qPCR analysis of neuronal differentiation marker *SNAP25*, *CHGA*, *SYN3* mRNA expression in SK-N-BE(2) cells treated with control (Ctrl_CM) or SLIT3-containing (SLIT3_CM) conditioned medium for 72 h. Data were presented as mean ± SD (n = 3, Student's t-test, ***p < 0.001, ****p < 0.0001). **I** RT-qPCR analysis of neuronal differentiation marker *SNAP25*, *CHGA*, *SYP* mRNA expression in SK-N-SH cells treated with Ctrl (Ctrl_CM) or SLIT3 (SLIT3_CM) conditioned medium for 72 h. Data were presented as mean ± SD (n = 3, Student's t-test, **p < 0.01, ***p < 0.001). **J** Schematic diagram showing the experimental design of doxycycline-inducible cell lines coculture. **K**, **L** Western blot analysis of doxycycline-induced SLIT3-3F expression upon 1 μg/mL doxycycline treatment for 48 h in donor cell lines (TRE-3F, pLVX-TRE-3Flag; TRE-SLIT3-3F, pLVX-TRE-SLIT3-3Flag) derived from SK-N-SH cells **(K)** and SK-N-BE(2) cells **(L)**. **M** Western blot analysis of neuronal differentiation marker pTrkA in cocultured SK-N-SH and SK-N-BE(2) recipient cells

To further investigate the effects of SLIT3 on neuroblastoma cells, we first treated neuroblastoma cell lines with SLIT3 recombinant protein and monitored changes in cell morphology. Treatment with 200 ng/mL SLIT3 for 72 h significantly increased the length of neurite outgrowth in SK-N-BE(2) and SK-N-SH cells (Fig. 4A, B). At the protein level, SLIT3 treatment upregulated neuronal differentiation markers including phosphorylated-TrkA (pTrkA), MAP2, and SNAP25 in SK-N-BE(2) and Kelly cells (Fig. 4C). Additionally, SLIT3 treatment enhanced the mRNA expression level of neuronal differentiation markers, including *SNAP25*, *CHGA*, *SYN3* in SK-N-BE(2) cells and *SNAP25*, *CHGA*, *SYP* in SK-N-SH cells (Fig. 4D, E).

To further confirm the function in multiple in vitro models, we established SLIT3-expressing (pLenti6-SLIT3-3Flag, SLIT3-3F) and control vector-containing (pLenti6-3Flag, Ctrl-3F) 293T stable cell lines for conditioned medium production (Fig. 4F). Neuroblastoma cells were cultured with the conditioned medium supplementation for 72 h, followed by analysis of neuronal differentiation markers. Consistent with the observations in recombinant protein treatment, cells treated with SLIT3-containing conditioned medium showed elevated expression of neuronal differentiation markers at both protein and transcriptional levels compared with control conditioned medium treated cells (Fig. 4G-I).

To better mimic the intratumoral effects of SLIT3-expressing subpopulation within the tumor microenvironment, we established a doxycycline-inducible coculture system for SLIT3-expressing donor cells and wild type recipient cells (Fig. 4J). In this coculture system, we mixed 30% donor cells (TRE-3F control vector or TRE-SLIT3-3F vector containing cells) with 70% mNeonGreen (mNG)-labeled wild-type recipient cells, and maintained the coculture for 5 days with continuous supplementation of 1 μg/mL doxycycline. Doxycycline-inducible SLIT3 expression in donor cell lines was confirmed by Western blot analysis (Fig. 4K, L). After coculture, mNG-labeled recipient cells (Top 30% mNG⁺) were then isolated by fluorescence-activated cell sorting (FACS) for differentiation validation. Notably, recipient cells that were cocultured with TRE-SLIT3-3F donors showed enhanced pTrkA protein levels relative to cells cocultured with TRE-3F donors (Fig. 4M). Collectively, these results demonstrated the capacity of SLIT3 to induce neuroblastoma cell differentiation in vitro.

### SLIT3 suppresses tumor growth and promotes neuroblastoma differentiation in vivo

**Fig. 5:**
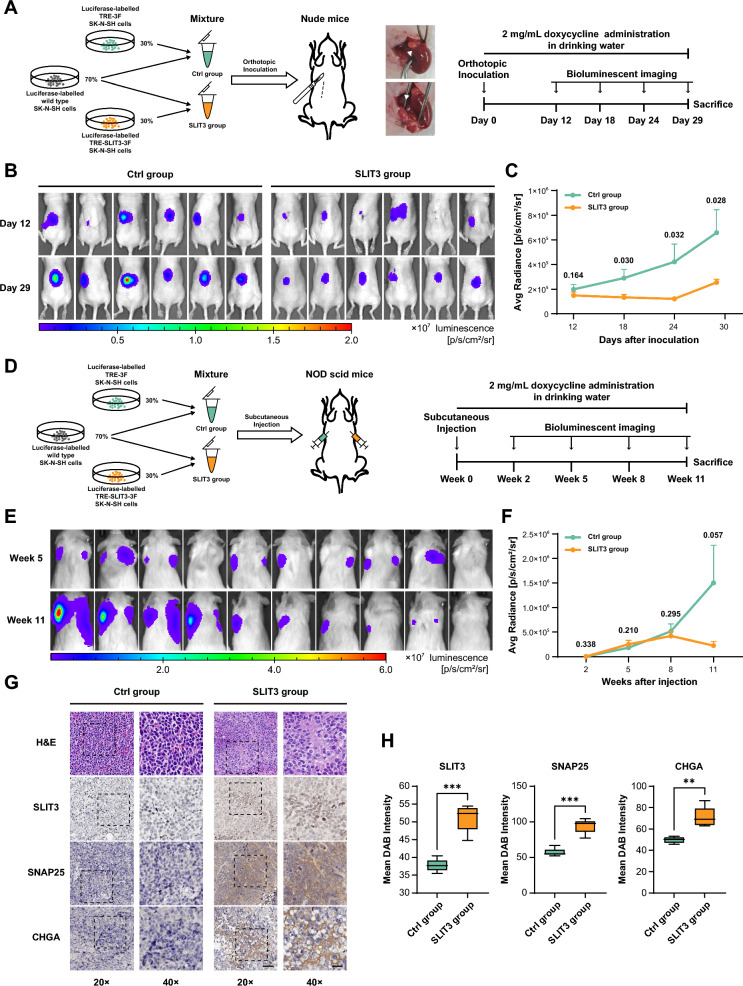
SLIT3 suppresses tumor growth and promotes neuroblastoma differentiation in vivo. **A** Schematic workflow illustrating the experimental design of orthotopic adrenal xenograft model. **B** Representative IVIS images of orthotopic adrenal xenografts in mice from Ctrl and SLIT3 groups at early (Day 12) and late stage (Day 29). **C** Quantification of bioluminescence signals at the presented days. Statistical significance between Ctrl group (n = 6) and SLIT3 group (n = 6) was determined by Student's t-test at each time point. Mean ± SEM. **D** Schematic workflow illustrating the experimental design of subcutaneous xenograft model. **E** Representative IVIS images of subcutaneous xenografts in mice at early (Week 5) and late stage (Week 11). **F** Quantification of bioluminescence signals at the presented weeks. Statistical significance between signals from Ctrl group (n = 10) and SLIT3 group (n = 10) was determined by Student's t-test at each time point. Mean ± SEM. **G** Representative images of H&E staining and immunohistochemical staining for SLIT3, SNAP25, and CHGA in subcutaneous xenograft tumor sections. Scale bars: 50 μm (20 ×) and 25 μm (40 ×). **H** Quantification of the DAB signal intensity in immunohistochemical staining of SLIT3, SNAP25, and CHGA. Statistical analyses were performed using data collected from 5 random fields of view for each protein per group. Student's t-test, **p < 0.01, ***p < 0.001

To investigate the in vivo differentiation effects, we established an orthotopic adrenal xenograft model by combining our doxycycline-inducible coculture system with a published neuroblastoma orthotopic adrenal xenograft model [52]. Briefly, luciferase-labelled SK-N-SH donor cells (TRE-3F or TRE-SLIT3-3F) were mixed with luciferase-labelled wild-type recipient cells at a ratio of 3:7, and the cell mixture was orthotopically implanted into the left adrenal gland of nude mice via surgery (Fig. 5A). To induce SLIT3 expression in the xenografts, mice were administered doxycycline (2 mg/mL) in their drinking water immediately following surgery. Bioluminescence signals revealed a progressive divergence in tumor growth between groups. By day 29, the SLIT3-expressing group exhibited significant tumor growth suppression compared to the Ctrl group (Fig. 5B, C), demonstrating the in vivo capacity of SLIT3 to suppress tumor growth.

To further validate these findings, subcutaneous xenograft models were established to evaluate SLIT3 effects in NOD-SCID mice (Fig. 5D). Tumor cell mixtures for xenograft implantation were prepared as in the previous orthotopic adrenal xenograft model, and implanted subcutaneously into the right and left flanks of NOD-SCID mice for SLIT3 and Ctrl groups, respectively. Mice were administered 2 mg/mL doxycycline in drinking water immediately after xenograft implantation to induce SLIT3 expression. By week 11, the SLIT3-expressing tumors showed a reduced growth trend compared with the Ctrl group (Fig. 5E, F). These results suggested that SLIT3 expression in 30% of the tumor cell population is capable of achieving moderate tumor growth suppression, consistent with our observations in the orthotopic model.

Histological analysis of tumor sections from subcutaneous xenograft models was then performed to validate the pathological changes. Tumors from Ctrl group were predominantly composed of small round blue cells with high nuclear-to-cytoplasmic ratios, similar to the pathological feature of undifferentiated neuroblastoma [53] (Fig. 5G, H&E panel). In contrast, tumors from SLIT3 group displayed enriched intercellular matrix with occurring neuropil-like structures, together with expanded, structured nucleoli, indicating a higher differentiation status (Fig. 5G, H&E panel). Immunohistochemical analysis confirmed the SLIT3 expression and demonstrated the significantly elevated expression of neuronal differentiation markers SNAP25 and CHGA in SLIT3 group tumors compared with Ctrl group (Fig. 5G, H). Collectively, these results suggested that SLIT3 expression in a subset of tumor cells was sufficient to suppress tumor growth and promote tumor differentiation in vivo.

### PLCβ/PKC signaling mediates SLIT3-induced neuroblastoma cell differentiation

**Fig. 6:**
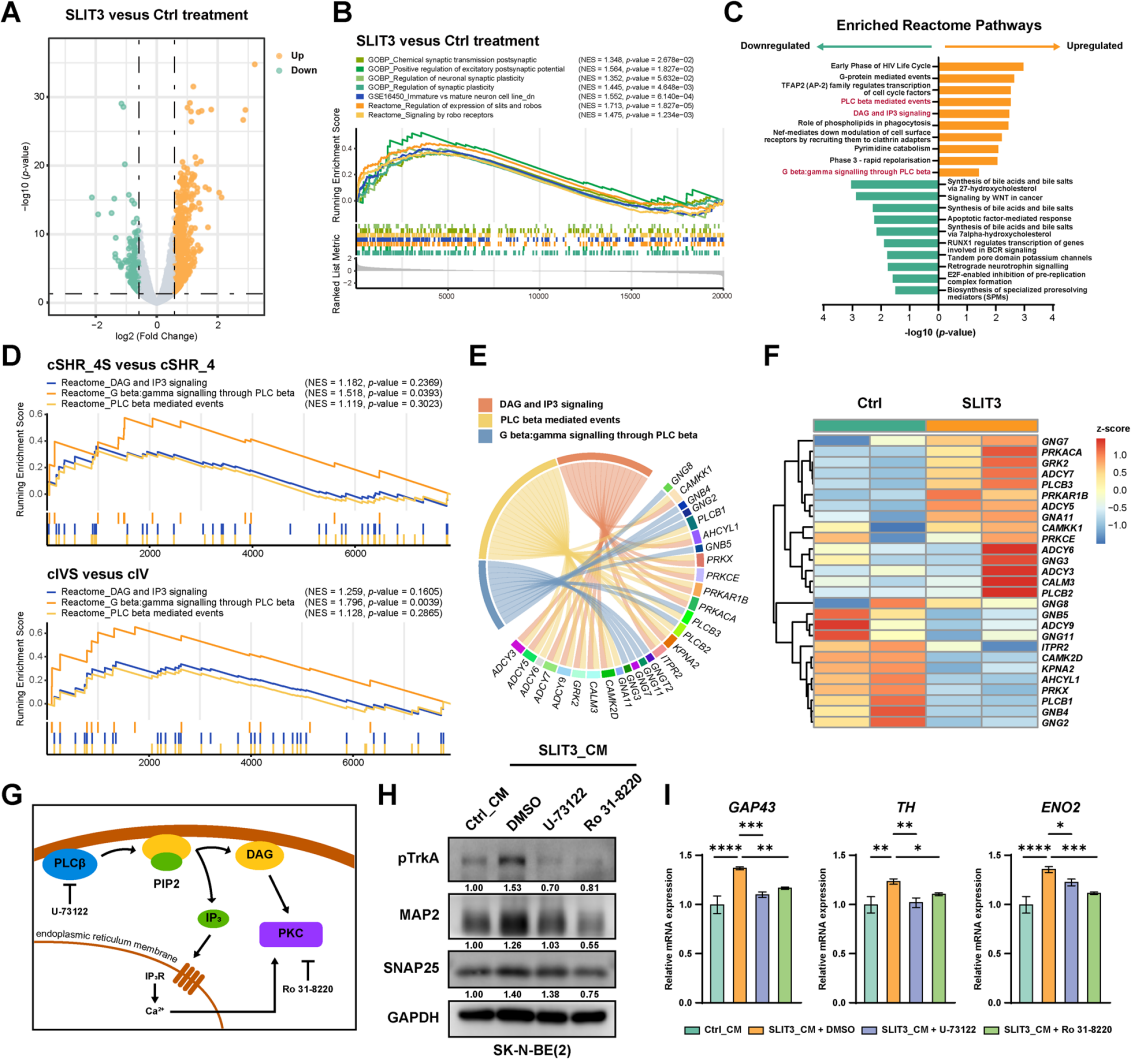
PLCβ/PKC signaling mediates SLIT3-induced neuroblastoma cell differentiation. **A** Volcano plot displaying DEGs comparing cells treated with or without 200 ng/mL SLIT3 recombinant protein for 72 h. Upregulated genes are colored in yellow and downregulated genes are colored in green. **B** GSEA on the MSigDB database revealed significant enrichment of SLIT-ROBO signaling gene sets and mature neuronal synaptic function gene sets upon SLIT3 treatment **C** GSEA analysis shows top 10 enriched pathways from Reactome database in SLIT3-treated group compared with Ctrl group. Upregulated and downregulated pathways are colored in yellow and green, respectively. **D** PLCβ/DAG/IP3 signaling-related gene sets enriched in Stage 4S sample cells compared with Stage 4 sample cells within cluster cSHR (upper panel), and consistently enriched in cluster cIVS compared with cluster cIV (lower panel). **E** Chord diagram showing gene composition of PLCβ/DAG/IP3 signaling-related gene sets. **F** Heatmap showing expression of common genes from PLCβ/DAG/IP3 signaling-related gene sets in SLIT3-treated versus Ctrl groups. **G** A brief overview of PLCβ/DAG/IP3/PKC signaling cascade. Upon activation, membrane-bound PLCβ catalyzes PIP2 hydrolysis into DAG and IP3. DAG directly activates PKC, while free IP3 binds to IP3R receptors on endoplasmic reticulum membrane, triggering calcium release and further activating PKC. **H** Western blot analysis of neuronal differentiation markers pTrkA, MAP2, SNAP25 in SK-N-BE(2) cells treated with 1 μM U-73122, or 2 μM Ro 31–8220 for 72 h in the presence of Ctrl or SLIT3 conditioned medium. **I** RT-qPCR analysis of neuronal differentiation markers *GAP43*, *TH*, and *ENO2* mRNA expression in SK-N-BE(2) cells treated with 1 μM U-73122, or 2 μM Ro 31–8220 for 72 h in the presence of Ctrl or SLIT3 conditioned medium. Data were presented as mean ± SD (n = 3, one-way ANOVA, *p < 0.05, **p < 0.01, ***p < 0.001, ****p < 0.0001)

To elucidate the downstream mechanisms mediating SLIT3-induced differentiation, we performed bulk RNA-seq on SK-N-BE(2) cells treated with or without 200 ng/mL recombinant SLIT3 protein for 72 h. Among 27,235 detected genes, we identified 872 upregulated and 319 downregulated genes in SLIT3 treatment group (|FC|> 1.5, *p* < 0.05, Fig. 6A). Mature neuronal synaptic function gene sets and SLIT-ROBO signaling gene sets were enriched in SLIT3-treated group (Fig. 6B). These data expectedly confirmed the effective activation of SLIT-ROBO signaling and neuronal differentiation programs upon SLIT3 treatment.

GSEA analysis on Reactome database revealed that phospholipase C beta (PLCβ) and diacylglycerol/inositol trisphosphate (DAG/IP3) signaling pathways were enriched in the top of upregulated Reactome pathways (Fig. 6C). These pathways showed similar enrichment patterns when comparing Stage 4S cells with Stage 4 cells within cluster cSHR, or cluster cIVS compared with cluster cIV, indicating that they were similarly activated in cIVS and cIVS associated tumor cells within Stage 4S tumor (Fig. 6D). Pearson correlation analysis on gene set scores also demonstrated the positive correlations between PLCβ/DAG/IP3 related pathways and SLIT-ROBO signaling-related pathways with data from neuroblastoma cohorts (Figure S3 A, B). Additionally, PLCβ/DAG/IP3 related pathways also displayed positive correlations with Neural crest cell differentiation pathway (Figure S3 C, D). These correlations suggested the potential involvement of PLCβ/DAG/IP3 signaling in SLIT-ROBO-mediated neuronal differentiation. Kaplan–Meier survival analysis revealed that patients with higher PLCβ/DAG/IP3 gene set scores exhibited significantly improved overall survival compared to those with low scores (Figure S4 A, B). To evaluate the diagnostic potential of these pathway signatures, we performed receiver operating characteristic (ROC) curve analysis for discriminating Stage 4S from Stage 4 neuroblastoma samples in Kocak, SEQC, and Cangelosi cohorts independently. Both PLCβ/DAG/IP3 related pathways and SLIT-ROBO signaling pathways demonstrated moderate discriminatory power (Figure S4 C), suggesting their potential utility as complementary diagnostic markers for Stage 4S tumor classification.

When looking into the gene composition of pathway gene sets, we found substantial overlap among these enriched pathways, suggesting their potential functional integration within a shared regulatory network (Fig. 6E, F). PLCβ and DAG/IP3 pathways constitute critical components of the phosphatidylinositol signaling system, with well-known functions in neuronal activity regulation and neurotransmitter signal transduction [54–56]. Upon activation, membrane-bound PLCβ hydrolyzes phosphatidylinositol 4,5-bisphosphate (PIP2) to generate two second messengers: DAG and IP3 [54, 57]. Cytoplasmic IP3 induces calcium release from the endoplasmic reticulum, leading to protein kinase C (PKC) activation, while membrane-bound DAG directly binds and activates PKC [58–62] (Fig. 6G).

To investigate whether PLCβ and its downstream PKC signaling pathway mediate SLIT3-induced neuroblastoma differentiation, we employed the PLCβ inhibitor U-73122 [63, 64] and PKC inhibitor Ro 31–8220 [65, 66] for signaling inhibition. Treatment with 1 μM U-73122 or 2 μM Ro 31–8220 for 72 h significantly attenuated SLIT3-induced neuronal differentiation in the presence of SLIT3 conditioned medium, as evidenced by decreased protein level of neuronal differentiation markers pTrkA, MAP2 and SNAP25 (Fig. 6H). Additionally, both U-73122 and Ro 31–8220 treatment inhibited mRNA expression levels of neuronal differentiation marker *GAP43*, *TH* and *ENO2* in SK-N-BE(2) cells under conditioned medium treatment (Fig. 6I). Collectively, our data suggested that PLCβ/PKC signaling plays an essential role in mediating SLIT3-induced neuroblastoma cell differentiation.

PKC signaling has been extensively reported to be activated in a hypoxic environment [67, 68]. In addition, hypoxia has been reported to play a role in regulating neuroblastoma differentiation, and Hypoxia-Inducible Factor 1-alpha (HIF1α) is capable of binding to the SLIT3 promoter [69–72]. These studies prompted us to further explore whether hypoxia contributes to the source of the SLIT3-expressing subpopulation. CoCl_2_ treatment and 1% O_2_ conditions were applied as hypoxic models in neuroblastoma cell lines, as previously described [73, 74]. Both treatments effectively activated the hypoxia response, evidenced by increased nuclear and total HIF1α protein levels as well as the elevated expression of HIF1α target genes (Figure S5 A-C). However, *SLIT3* expression decreased upon hypoxic conditions, and correlation analysis from neuroblastoma patient cohorts revealed no significant association between hypoxia markers and SLIT3 expression (Figure S5 D, E). Consistent with previous reports, *ENO2* and *TH* expression increased upon hypoxia treatment in both models (Figure S5 F) [69, 70, 73]. However, *SNAP25* and *GAP43* expression decreased under hypoxic conditions (Figure S5 G). In hypoxic groups, SNAP25 protein level was consistently reduced in both cell lines, and pTrkA was significantly inhibited in Kelly cells, while MAP2 expression remained mainly unchanged (Figure S5 H). These findings indicated that SLIT3-induced differentiation is likely representing a distinct differentiation state from that induced by hypoxia, and showed that hypoxia exerts multifaceted effects on neuroblastoma differentiation. Nevertheless, the shared upregulated genes suggested that hypoxia- and SLIT3-mediated differentiation pathways may partially overlap, potentially sharing certain downstream mechanisms. The detailed molecular crosstalk between hypoxia- and SLIT3-induced differentiation warrants further comprehensive investigation.

## Discussion

**Fig. 7:**
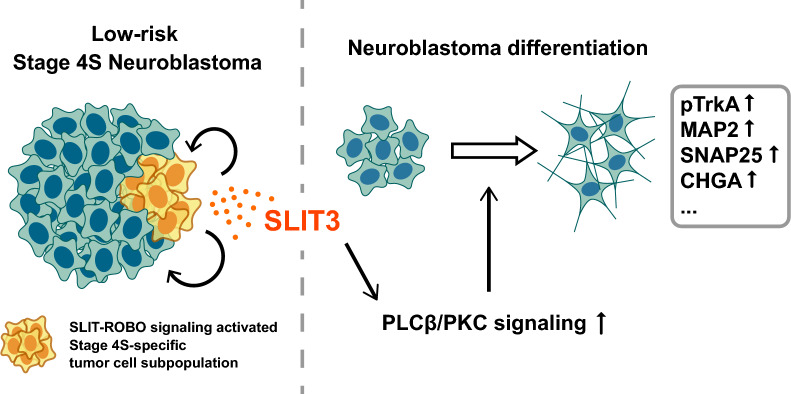
Schematic model of SLIT3-mediated neuroblastoma differentiation. Stage 4S-specific tumor cell subpopulation-derived SLIT3 supports intratumoral communication (left). PLCβ/PKC signaling mediates SLIT3-induced differentiation in neuroblastoma cells (right)

In this study, through comprehensive analysis of snRNA-seq data derived from Stage 4S and Stage 4 neuroblastoma, we uncovered a novel mechanism mediating neuroblastoma differentiation. Our findings demonstrated that activation of SLIT-ROBO signaling represents a distinguishing feature of Stage 4S neuroblastoma. SLIT3, which is enriched in the Stage 4S-specific tumor cell subpopulation, acts as a potential differentiation inducer functioning through PLCβ/PKC signaling (Fig. 7). These discoveries provide mechanistic insights into spontaneous regression and reveal novel therapeutic targets for neuroblastoma treatment.

Neuroblastoma presents diverse clinical outcomes depending on tumor stage and molecular characteristics. Low-risk neuroblastoma, particularly INSS Stage 4S tumors, exhibits favorable outcomes with a high rate of spontaneous tumor regression, leading to the trend of therapy minimization [75, 76]. In contrast, high-risk cases remain challenging due to drug resistance and relapse even after multimodal combination therapy [8]. Although retinoic acid-induced differentiation therapy demonstrates initial efficacy in early treatment of high-risk neuroblastoma, its long-term effectiveness is limited, and the difficulties in controlling pediatric dosage frequently result in side effects [8, 77–79]. These limitations highlight the urgent need for novel molecular targets in differentiation therapy, particularly those that can induce sustained therapeutic responses with minimal side effects. In prior large-scale screening programs for pediatric neuroblastoma, numerous children were found to have highly differentiated residual neuroblastoma lesions without ever exhibiting any clinical symptoms of neuroblastoma [80–82]. This suggests that spontaneous regression of neuroblastoma represents a mechanism with minimal adverse effects, which holds promise for development into more efficient clinical therapies with reduced side effects. By analyzing the heterogeneous cellular composition inside Stage 4S neuroblastoma which exhibits the highest likelihood of spontaneous regression, we discovered an intrinsic program regulating neuroblastoma differentiation through intratumoral crosstalk on SLIT-ROBO signaling. This mechanism naturally exists within neuroblastoma and can be considered as a differentiation-inducing mechanism accepted by the tumor, representing a potential target for differentiation-inducing therapy with minimal side effects.

Furthermore, neuroblastoma predominantly affects infants with immature physiological functions, particularly in hepatic detoxification and hematopoiesis, making them especially vulnerable to treatment-related complications [83–85]. The absence of specific molecular biomarkers for Stage 4S diagnosis, together with the common feature of being disseminated in both high-risk Stage 4 and low-risk Stage 4S disease, necessitates the reliance on age-specific metastatic patterns for diagnosis [3]. This diagnostic challenge may lead to confusion between Stage 4S and Stage 4 disease and inappropriate treatment intensity in the early stage of tumor progression [84, 86, 87]. Therefore, comprehensive investigation of Stage 4S tumor biological characteristics is essential for accurate early-stage diagnosis. In our study, we identified elevated SLIT3 expression in a Stage 4S-specific tumor cell subpopulation, suggesting its potential as an auxiliary diagnostic marker for Stage 4S, and an indicator of spontaneous regression. This finding helps to enhance the diagnostic accuracy of low-risk tumors, thereby reducing unnecessary therapeutic interventions.

Various malignancies, including melanoma and lymphoma, show reports of spontaneous regression, but the highest incidence is observed in Stage 4S neuroblastoma, contributing to its favorable clinical outcomes [11, 12, 88]. While the underlying mechanisms of spontaneous regression remain incompletely understood, current hypotheses emphasize the roles of neurotrophic pathway activation, immune clearance, and tumor cell apoptosis [13]. In neuroblastoma, tumor differentiation frequently accompanies spontaneous regression [89, 90]. The identified spontaneous regression-like program in Stage 4S tumor progressing toward differentiation further confirms that differentiation represents a critical step in the spontaneous regression process. Moreover, the activation of neurotrophic factor receptor TrkA and its downstream pathways in Stage 4S neuroblastoma also supports the association between differentiation and spontaneous regression [13]. Our study revealed an intrinsic differentiation-inducing mechanism within Stage 4S tumors, where SLIT3 conveys intercellular communication, activating downstream PLCβ/PKC signaling. These findings provide novel mechanistic insights into neuroblastoma spontaneous regression and advance potential clinical applications. Notably, differentiated neuroblastoma cells exhibit elevated expression of immunogenic molecules, potentially contributing to enhanced immune recognition and clearance [91, 92]. Targeting these intrinsic differentiation regulatory mechanisms in high-risk neuroblastoma may provide an opportunity to activate immune clearance of tumor cells, guiding malignant tumors toward a more benign and self-limiting state, and improving survival outcomes in pediatric patients.

SLIT-ROBO signaling plays a primary role in axon guidance, while also mediating differentiation across neurons and other organs [47, 48, 93]. Recent studies have reported the anti-tumor effects including suppression of tumor cell proliferation and regulation of antitumor immunity [94, 95]. These reports suggest the possibility of simultaneously inducing tumor cell differentiation and immune cell recruitment, facilitating tumor clearance and potentially contributing to spontaneous regression by targeting SLIT-ROBO signaling. Given that ROBO receptors lack intrinsic enzymatic activity in their intracellular domains, they predominantly mediate signal transduction through interactions with multiple adaptor molecules and diverse downstream signaling pathways [24, 96]. Moreover, since effective small molecule activators for ROBO receptors remain undeveloped, targeting the downstream signaling pathways activated by SLIT3 presents an available therapeutic strategy. Our study revealed that SLIT3 regulates tumor cell differentiation through the PLCβ/PKC signaling pathway, providing novel mechanistic insights and available therapeutic targets for clinical translation.

As another well-established factor activating PKC signaling, hypoxia has been reported to play a role in regulating neuroblastoma differentiation [67–69]. Different PKC isoforms are activated in a hypoxic environment depending on the cellular context. For instance, PKC-βII, γ, η, μ, and λ are upregulated in response to acute hypoxia in brain microvessel endothelial cells, while PKC-ε and ζ expression are activated during the subsequent reoxygenation process [97]. In cardiomyocytes under chronic hypoxic conditions, PKC-δ is activated to facilitate adaptation to hypoxic environments and provide cardioprotection [98, 99]. Notably, a study has reported that PKC-δ is activated during retinoic acid-induced neuroblastoma cell differentiation and regulates MAP2 expression [100]. Additionally, PKC-α and PKC-ε have been reported to modulate noradrenaline function in human neuroblastoma cells [101, 102]. These studies indicate that different PKC isoforms may be involved in regulating distinct differentiation states. The shared upregulated genes observed following both SLIT3 and hypoxia treatment in our experimental results imply partially overlapping downstream mechanisms between hypoxia- and SLIT3-mediated differentiation. Overlapping PKC isoforms likely serve as the molecular foundation shared between hypoxia-induced and SLIT3-induced neuroblastoma differentiation, suggesting the potential of hypoxia to regulate SLIT3-induced differentiation. Future studies are needed to clarify the specific profile of PKC isoforms and the microenvironmental conditions through which hypoxia regulates neuroblastoma differentiation and how these interact with SLIT3-mediated pathways.

Despite these inspiring findings, several limitations exist in our study. The snRNA-seq data utilized in this study relied solely on the rare Stage 4S samples. Although Smart-seq2 protocol provides full-length transcript coverage, enabling the comprehensive detection of Stage 4S samples transcriptome, broader validation on clinical samples is necessary. The detailed mechanisms underlying the in vivo clearance of differentiated neuroblastoma cells remain to be fully elucidated, which may contribute to the ultimate tumor regression. Particularly, the potential involvement of immune cells in this process requires systematic examination. Additionally, the cellular origin of SLIT-ROBO-activated cells in Stage 4S tumors merits comprehensive investigation to elucidate the initiation of spontaneous regression. Further investigation into these aspects would enhance the understanding of the spontaneous regression phenomenon in neuroblastoma and facilitate the development of targeted therapeutic strategies for high-risk neuroblastoma patients.

## Conclusions

We identified and characterized a distinct tumor cell subpopulation and a spontaneous regression-like program within low risk INSS Stage 4S neuroblastoma tumors. SLIT-ROBO signaling pathway was activated in this subpopulation and correlated with improved patient survival. Our functional studies revealed that SLIT3, a key ligand in the SLIT-ROBO signaling, promotes tumor cell differentiation and suppresses tumor growth via intratumoral crosstalk. Mechanistically, SLIT3 induces tumor cell differentiation through the PLCβ/PKC signaling. Our findings provided a novel mechanism underlying spontaneous regression phenomenon in Stage 4S neuroblastoma, and offered potential therapeutic targets for differentiation-based therapy in high-risk neuroblastoma.

## Supplementary Information

Additional file 1. Supplementary Figure Legends

Additional file 2. Supplementary Figure 1. Identifying tumor cell clusters based on unsupervised clustering analysis

Additional file 3. Supplementary Figure 2. Survival analysis based on SLIT-ROBO signaling related gene sets scores

Additional file 4. Supplementary Figure 3. Correlation analysis of gene set scores across multiple neuroblastoma cohorts

Additional file 5. Supplementary Figure 4. Clinical significance of PLCβ/DAG/IP3 signaling in neuroblastoma cohorts

Additional file 6. Supplementary Figure 5. Hypoxia effects on SLIT3 expression and neuroblastoma differentiation

Additional file 7. Supplementary Table S1. Primers for RT-qPCR analysis of neuronal differentiation marker genes and internal control gene

## Data Availability

The snRNA-seq datasets supporting the conclusions of this article are part of previously published data obtained from the Synapse projects: syn22302605, which were available in the Synapse repository (https://www.synapse.org) upon reasonable request. Public neuroblastoma cohorts for differential expression analysis, gene set scoring and survival analysis were available from R2 platform (https://hgserver2.amc.nl/cgi-bin/r2/main.cgi).
