# Supplementary material for: SLIT3-mediated intratumoral crosstalk induces neuroblastoma differentiation via a spontaneous regression-like program
Source: J Transl Med. 2025 May 30;23:598. doi: 10.1186/s12967-025-06621-0 (PMC12123822; doi:10.1186/s12967-025-06621-0)
Supplement: Supplementary file 1 — Additional file 1. Supplementary Figure Legends [file 12967_2025_6621_MOESM1_ESM.pdf]

Supplementary Figure 1: Identifying tumor cell clusters based on unsupervised clustering analysis. (A) tSNE visualization of single-nucleus colored by disease stage. **(B)** tSNE visualization of single-nucleus colored by sample origin. **(C)** tSNE visualization of single-nucleus colored by unsupervised clustering result. **(D)** Cluster composition of sample information. Top left panel shows the distribution of sample origins across the identified clusters. Top right panel displays the proportion of disease stages within each cluster. Bottom panel illustrates the relative abundance of each cluster in samples from Stage 4 and Stage 4S. **(E)** Heatmap showing SingleR-based cell type annotation scores across the identified 12 clusters. **(F)** Bubble plot showing expression patterns of cell type marker genes across the identified 12 clusters. **(G)** Heatmap showing CNV intensity patterns across chromosomes for each cluster. Clusters c1, c7, c8, c11, and c12 served as diploid reference cells. **(H)** Violin plots displaying the distribution of mean CNV scores across the 12 clusters.

Supplementary Figure 2: Survival analysis based on SLIT-ROBO signaling related gene sets scores. Kaplan-Meier analysis of overall survival in neuroblastoma patients from independent cohorts: Kocak (n=476), SEQC (n=498), Cangelosi (n=786), Oberthuer (n=251), Versteeg (n=88). Patients were stratified by gene set scores for **(A)** "Axon guidance mediated by SLIT-ROBO", **(B)** "Signaling by ROBO receptors", and **(C)** "Roundabout signaling pathway", and classified into high (red) and low (blue) expression groups based on z-scores of respective gene sets. Statistical significance was determined by log-rank test.

Supplementary Figure 3: Correlation analysis of gene set scores across multiple neuroblastoma cohorts. Pearson correlation analysis of gene set scores derived from multiple bulk RNA-seq datasets in R2 platform: **(A)** "PLC-beta mediated events" and "Axon guidance mediated by SLIT-ROBO", **(B)** "DAG and IP3 signaling" and "Axon

guidance mediated by SLIT-ROBO", **(C)** "PLC-beta mediated events" and "Neural crest cell differentiation", and **(D)** "DAG and IP3 signaling" and "Neural crest cell differentiation". All gene set scores were presented as z-scores.

Supplementary Figure 4: Clinical significance of PLCβ/DAG/IP3 signaling in neuroblastoma cohorts. Kaplan-Meier analysis of overall survival in neuroblastoma patients from independent cohorts: Kocak (n=476), SEQC (n=498), Cangelosi (n=786), Oberthuer (n=251), Versteeg (n=88), and NRC (n=276). Patients were divided into high (red) and low (blue) expression groups based on gene set scores for "PLC-beta mediated events" **(A)** and "DAG and IP3 signaling" **(B)**. Statistical significance was determined by log-rank test. **(C)** ROC curves evaluating the discriminative power of gene set scores between Stage 4S and Stage 4 tumors in Kocak, SEQC, and Cangelosi cohorts independently. Statistical significance was determined by Wilcoxon Rank Sum test.

Supplementary Figure 5: Hypoxia effects on SLIT3 expression and neuroblastoma differentiation. (A) Immunofluorescence staining of HIF1α in cells treated with or without 100 μM $CoCl_2$ for 6 h, or maintained in 21% O_2 or 1% O_2 for 6 h. DAPI is in blue and HIF1α is in green. Scale bar: 20 μm. **(B)** RT-qPCR analysis of hypoxia markers *VEGFA* and *GLUT1* mRNA expression in cells treated with or without 100 μM $CoCl_2$ for 6 h, or maintained in 21% O_2 or 1% O_2 for 6 h. **(C)** Western blot analysis of hypoxia marker HIF1α in cells treated with or without 100 μM $CoCl_2$ for 6 h, or maintained in 21% O_2 or 1% O_2 for 6 h. **(D)** RT-qPCR analysis of *SLIT3* mRNA expression in cells treated with or without 100 μM $CoCl_2$ for 24 h, or maintained in 21% O_2 or 1% O_2 for 24 h. **(E)** Pearson correlation analysis of SLIT3 expression and hypoxia markers expression derived from neuroblastoma cohort data in R2 platform. Gene expression was presented as Log2 z-score. **(F)** RT-qPCR analysis of neuronal differentiation

markers *ENO2* and *TH* mRNA expression in cells treated with or without 100 μM $CoCl_2$ for 24 h, or maintained in 21% O_2 or 1% O_2 for 24 h. **(G)** RT-qPCR analysis of neuronal differentiation markers *SNAP25* and *GAP43* mRNA expression in cells treated with or without 100 μM $CoCl_2$ for 24 h, or maintained in 21% O_2 or 1% O_2 for 24 h. **(H)** Western blot analysis of neuronal differentiation markers pTrkA, MAP2, SNAP25 in cells treated with or without 100 μM $CoCl_2$ for 24 h, or maintained in 21% O_2 or 1% O_2 for 24 h. Experimental data for statistical analysis were presented as mean ± SD (n = 3, Student's t-test, *$p < 0.05$, **$p < 0.01$, ***$p < 0.001$, ****$p < 0.0001$).
