# Supplementary figures and images for: SLIT3-mediated intratumoral crosstalk induces neuroblastoma differentiation via a spontaneous regression-like program
Source: J Transl Med. 2025 May 30;23:598. doi: 10.1186/s12967-025-06621-0 (PMC12123822; doi:10.1186/s12967-025-06621-0)

**A**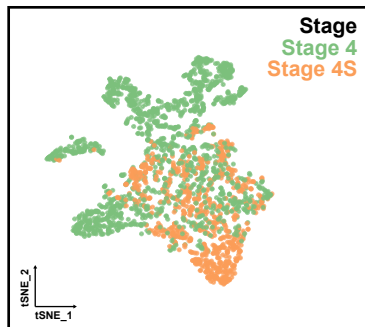**B**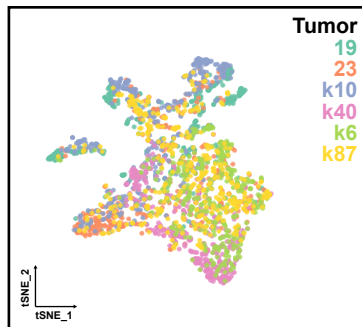**C**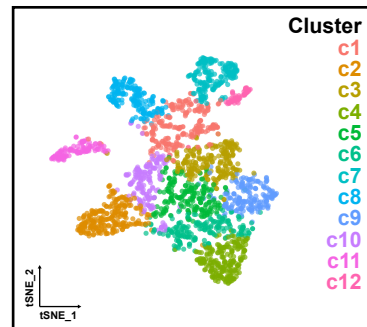**D**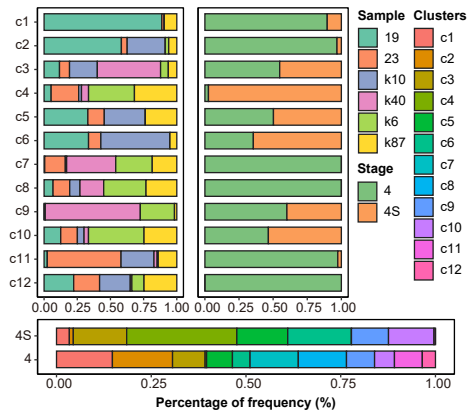**E**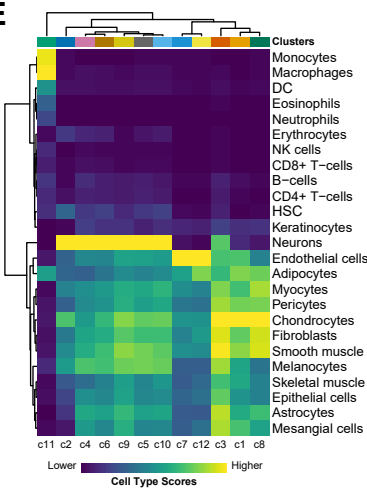**F**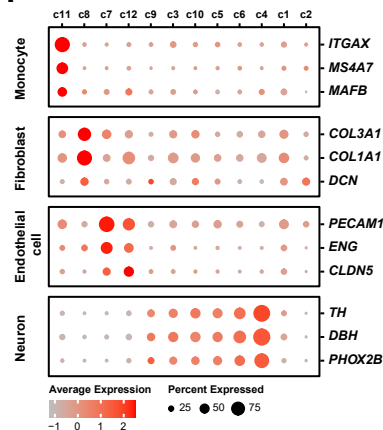**G**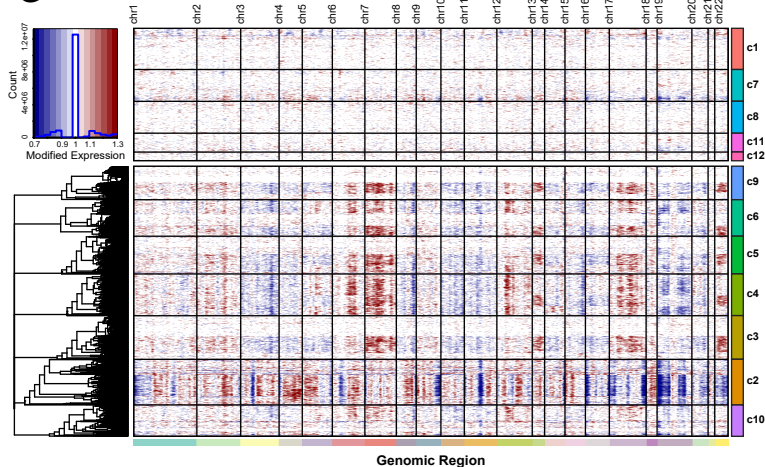**H**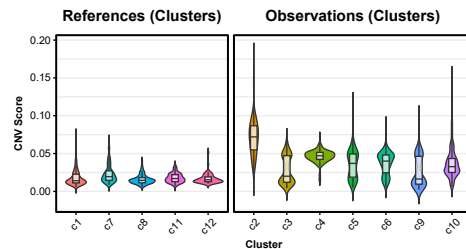

Supplement: Supplementary file 2 — Additional file 2. Supplementary Figure 1. Identifying tumor cell clusters based on unsupervised clustering analysis [file 12967_2025_6621_MOESM2_ESM.pdf]

**A**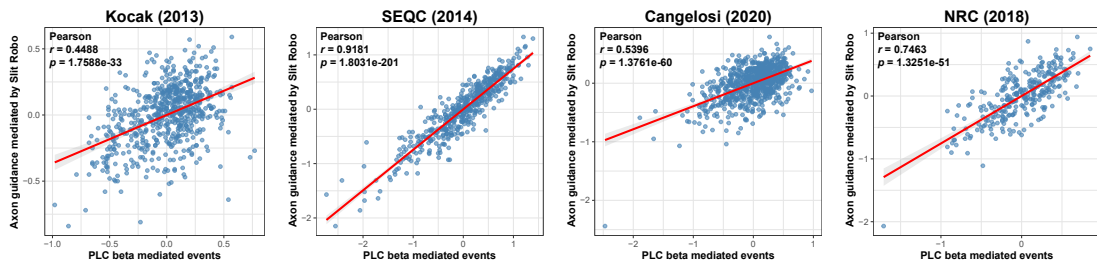**B**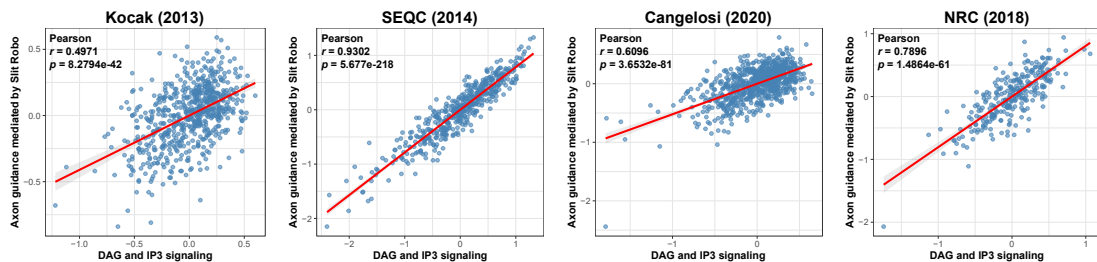**C**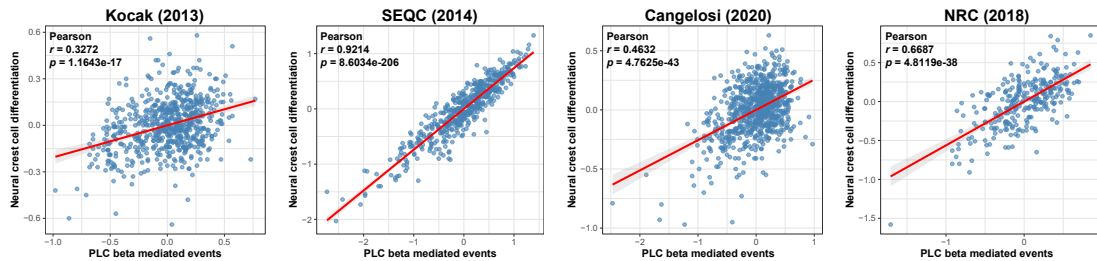**D**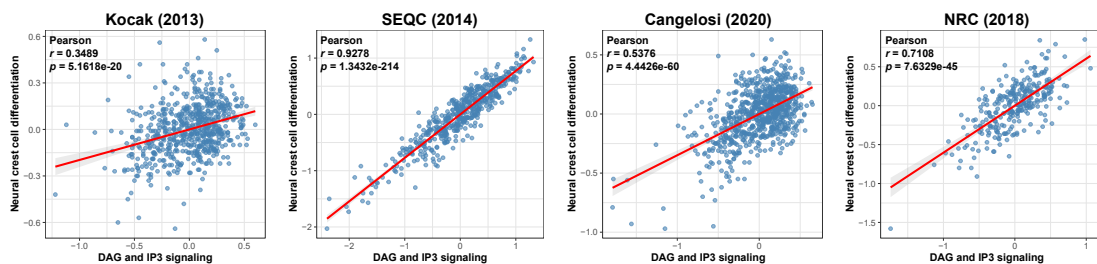

Supplement: Supplementary file 4 — Additional file 4. Supplementary Figure 3. Correlation analysis of gene set scores across multiple neuroblastoma cohorts [file 12967_2025_6621_MOESM4_ESM.pdf]

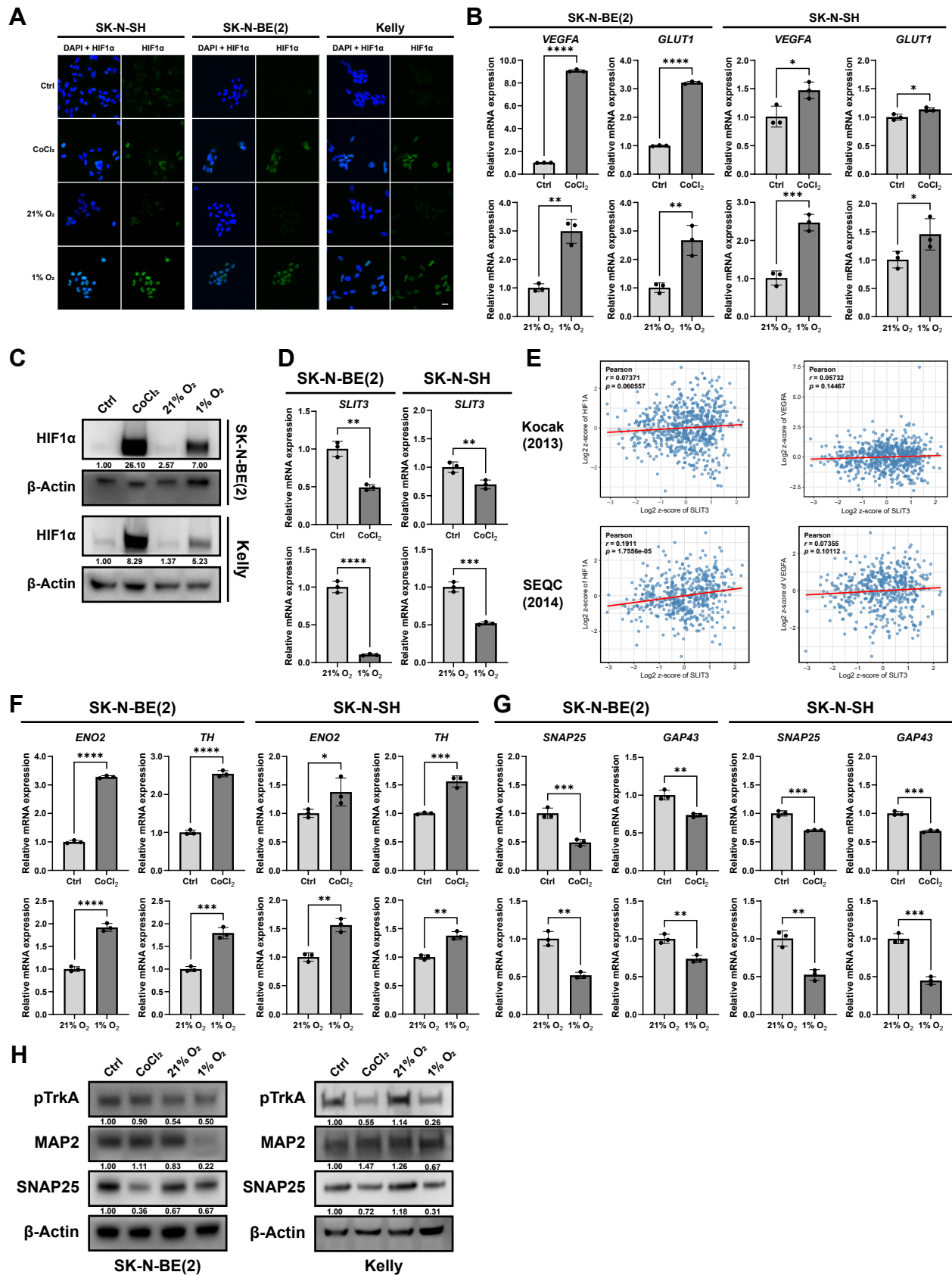

Supplement: Supplementary file 6 — Additional file 6. Supplementary Figure 5. Hypoxia effects on SLIT3 expression and neuroblastoma differentiation [file 12967_2025_6621_MOESM6_ESM.pdf]
