# Supplementary material for: SLIT3-mediated intratumoral crosstalk induces neuroblastoma differentiation via a spontaneous regression-like program
Source: J Transl Med. 2025 May 30;23:598. doi: 10.1186/s12967-025-06621-0 (PMC12123822; doi:10.1186/s12967-025-06621-0)
Supplement: Supplementary file 3 — Additional file 3. Supplementary Figure 2. Survival analysis based on SLIT-ROBO signaling related gene sets scores [file 12967_2025_6621_MOESM3_ESM.pdf]

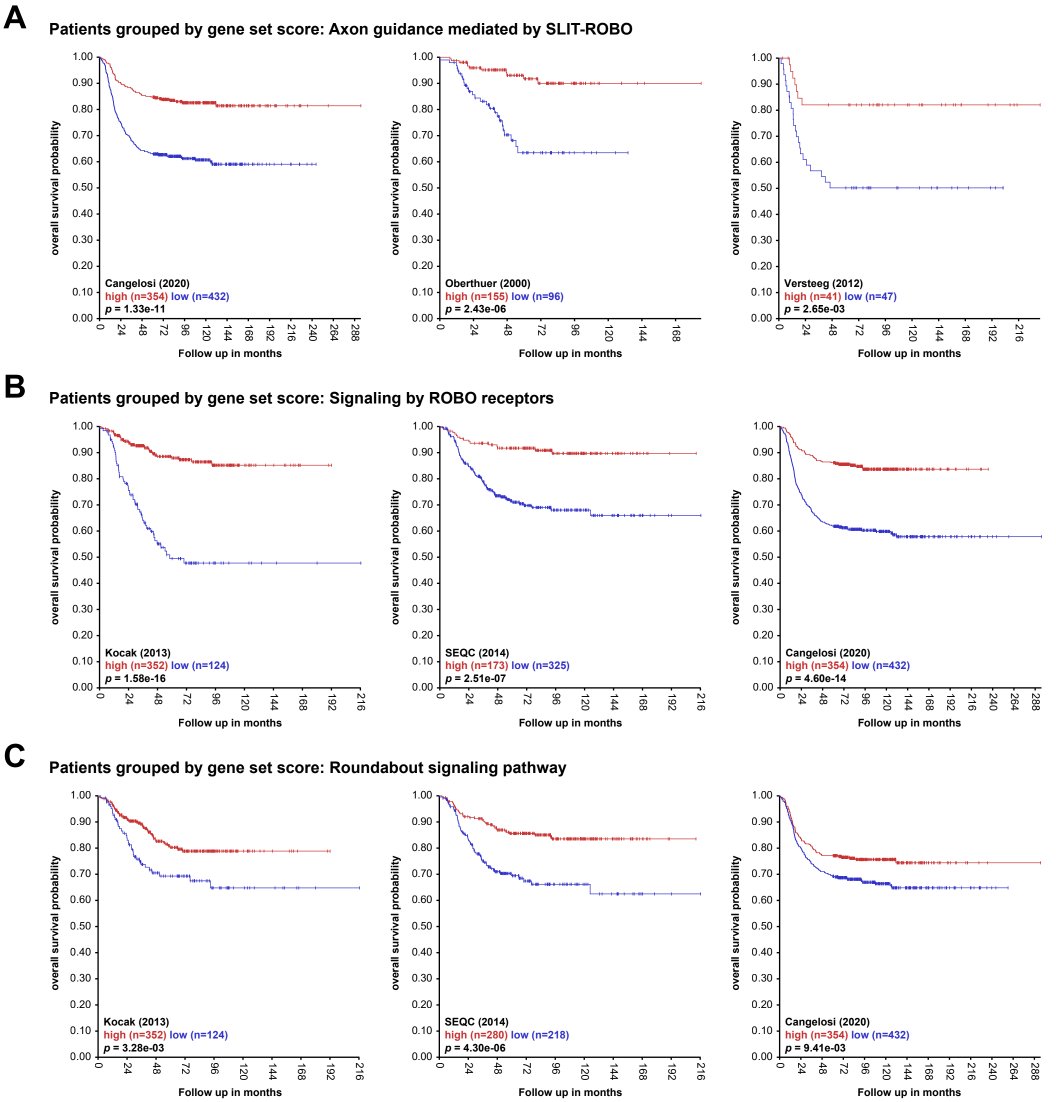
A
Patients grouped by gene set score: Axon guidance mediated by SLIT-ROBO
Cangelosi (2020)
high (n=354) low (n=432)
p = 1.33e-11
Oberthuer (2000)
high (n=155) low (n=96)
p = 2.43e-06
Versteeg (2012)
high (n=41) low (n=47)
p = 2.65e-03
B
Patients grouped by gene set score: Signaling by ROBO receptors
Kocak (2013)
high (n=352) low (n=124)
p = 1.58e-16
SEQC (2014)
high (n=173) low (n=325)
p = 2.51e-07
Cangelosi (2020)
high (n=354) low (n=432)
p = 4.60e-14
C
Patients grouped by gene set score: Roundabout signaling pathway
Kocak (2013)
high (n=352) low (n=124)
p = 3.28e-03
SEQC (2014)
high (n=280) low (n=218)
p = 4.30e-06
Cangelosi (2020)
high (n=354) low (n=432)
p = 9.41e-03
overall survival probability
Follow up in months
