# Supplementary material for: SLIT3-mediated intratumoral crosstalk induces neuroblastoma differentiation via a spontaneous regression-like program
Source: J Transl Med. 2025 May 30;23:598. doi: 10.1186/s12967-025-06621-0 (PMC12123822; doi:10.1186/s12967-025-06621-0)
Supplement: Supplementary file 5 — Additional file 5. Supplementary Figure 4. Clinical significance of PLCβ/DAG/IP3 signaling in neuroblastoma cohorts [file 12967_2025_6621_MOESM5_ESM.pdf]

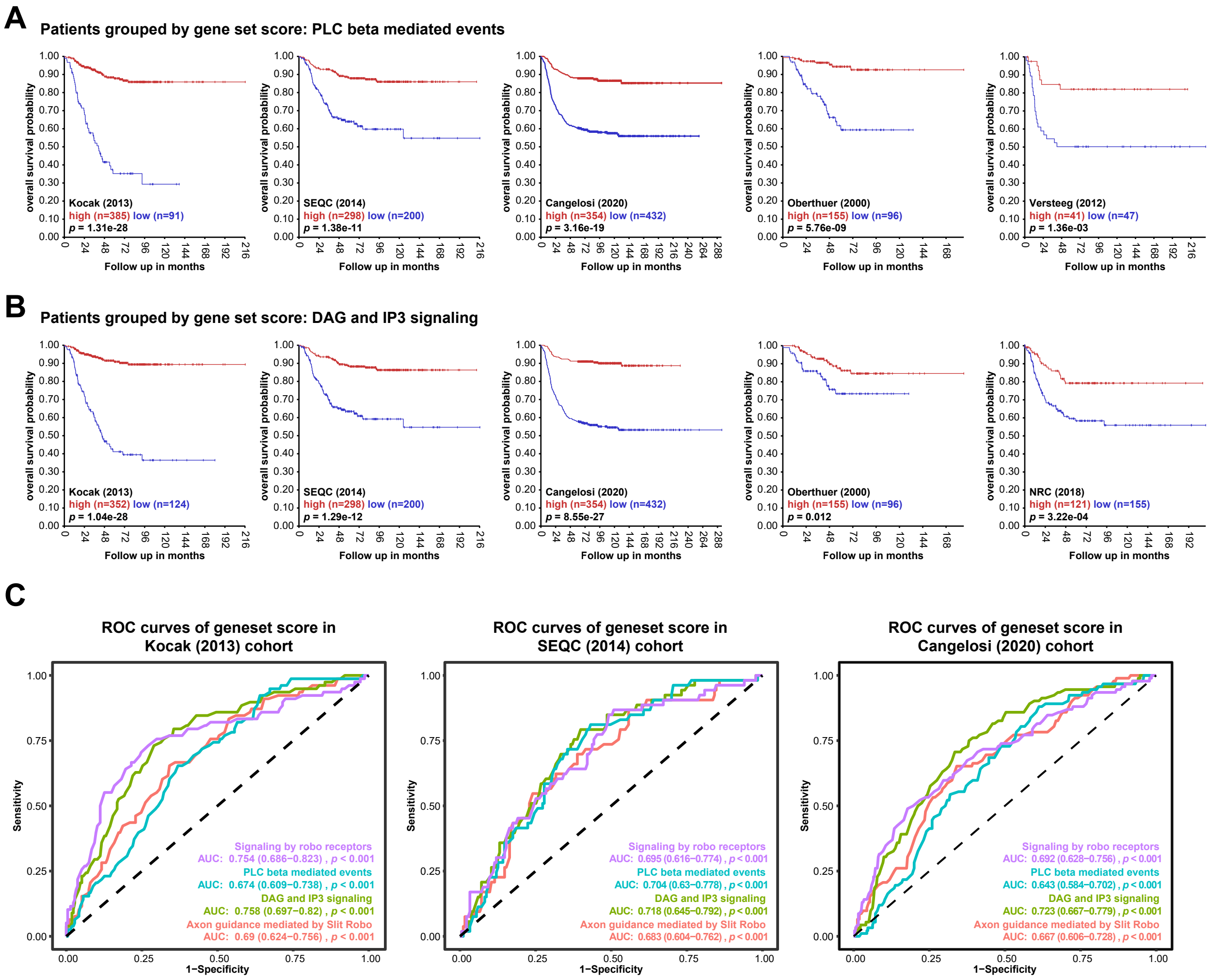
A
Patients grouped by gene set score: PLC beta mediated events
overall survival probability
Follow up in months
Kocak (2013)
high (n=385) low (n=91)
p = 1.31e-28
SEQC (2014)
high (n=298) low (n=200)
p = 1.38e-11
Cangelosi (2020)
high (n=354) low (n=432)
p = 3.16e-19
Oberthuer (2000)
high (n=155) low (n=96)
p = 5.76e-09
Versteeg (2012)
high (n=41) low (n=47)
p = 1.36e-03
B
Patients grouped by gene set score: DAG and IP3 signaling
overall survival probability
Follow up in months
Kocak (2013)
high (n=352) low (n=124)
p = 1.04e-28
SEQC (2014)
high (n=298) low (n=200)
p = 1.29e-12
Cangelosi (2020)
high (n=354) low (n=432)
p = 8.55e-27
Oberthuer (2000)
high (n=155) low (n=96)
p = 0.012
NRC (2018)
high (n=121) low (n=155)
p = 3.22e-04
C
ROC curves of geneset score in Kocak (2013) cohort
Sensitivity
1−Specificity
Signaling by robo receptors
AUC: 0.754 (0.686−0.823) , p < 0.001
PLC beta mediated events
AUC: 0.674 (0.609−0.738) , p < 0.001
DAG and IP3 signaling
AUC: 0.758 (0.697−0.82) , p < 0.001
Axon guidance mediated by Slit Robo
AUC: 0.69 (0.624−0.756) , p < 0.001
ROC curves of geneset score in SEQC (2014) cohort
Signaling by robo receptors
AUC: 0.695 (0.616−0.774) , p < 0.001
PLC beta mediated events
AUC: 0.704 (0.63−0.778) , p < 0.001
DAG and IP3 signaling
AUC: 0.718 (0.645−0.792) , p < 0.001
Axon guidance mediated by Slit Robo
AUC: 0.683 (0.604−0.762) , p < 0.001
ROC curves of geneset score in Cangelosi (2020) cohort
Signaling by robo receptors
AUC: 0.692 (0.628−0.756) , p < 0.001
PLC beta mediated events
AUC: 0.643 (0.584−0.702) , p < 0.001
DAG and IP3 signaling
AUC: 0.723 (0.667−0.779) , p < 0.001
Axon guidance mediated by Slit Robo
AUC: 0.667 (0.606−0.728) , p < 0.001
